# Morphological variation, phylogenetic relationships, and geographic distribution of the Baenidae (Testudines), based on new specimens from the Uinta Formation (Uinta Basin), Utah (USA)

**DOI:** 10.1371/journal.pone.0180574

**Published:** 2017-07-07

**Authors:** Heather F. Smith, J. Howard Hutchison, K. E. Beth Townsend, Brent Adrian, Daniel Jager

**Affiliations:** 1 Department of Anatomy, Midwestern University, Glendale, AZ, United States of America; 2 College of Veterinary Medicine, Midwestern University, Glendale, AZ, United States of America; 3 School of Human Evolution and Social Change, Arizona State University, Tempe, AZ, United States of America; 4 University of California Museum of Paleontology, University of California Berkeley, Berkeley, CA, United States of America; Royal Belgian Institute of Natural Sciences, BELGIUM

## Abstract

We described newly discovered baenid specimens from the Uintan North American Land Mammal Age (NALMA), in the Uinta Formation, Uinta Basin, Utah. These specimens include a partial skull and several previously undescribed postcranial elements of *Baena arenosa*, and numerous well-preserved shells of *B*. *arenosa* and *Chisternon undatum*. Baenids from the Uintan NALMA (46.5–40 Ma) are critical in that they provide valuable insight into the morphology and evolution of the diverse and speciose baenid family near the end of its extensive radiation, just prior to the disappearance of this clade from the fossil record. These Uintan specimens greatly increase the known variation in these late-surviving taxa and indicate that several characters thought to define these species should be reassessed. The partial cranium of *B*. *arenosa*, including portions of the basicranium, neurocranium, face, and lower jaw, was recently recovered from Uinta B sediments. While its morphology is consistent with known specimens of *B*. *arenosa*, we observed several distinct differences: a crescent-shaped condylus occipitalis that is concave dorsally, tuberculum basioccipitale that flare out laterally, and a distinct frontal-nasal suture. The current sample of plastral and carapacial morphology considerably expands the documented variation in the hypodigms of *B*. *arenosa* and *C*. *undatum*. Novel shell characters observed include sigmoidal extragular-humeral sulci, and small, subtriangular gular scutes. Subadult specimens reveal ontogenetic processes in both taxa, and demonstrate that diagnostic morphological differences between them were present from an early developmental age.

## Introduction

The Baenidae [1] were an extinct clade of North American freshwater river turtles with an extensive radiation spanning from the early Cretaceous to the middle Eocene [2–5]. More than 30 different baenid species have been recognized from the fossil record [6], all united by a common synapomorphic tendency to co‐ossify the shell and skull in adulthood. Baenids are the most abundant, diverse, and speciose Testudine family in the Cretaceous fossil record [2–3, 7], and persisted past the Cretaceous-Tertiary (K-T) Boundary, surviving the Cretaceous-Paleocene (K-Pg) mass extinction event, during which many other sauropsid taxa died out. The baenid clade finally disappeared entirely from the fossil record during the Eocene, with the last documented appearance of this prolific family occurring during the Uintan North American Land Mammal Age (NALMA; 46.5–40 Ma) [8–11]. An undocumented occurrence could also be present in the late Eocene (Duchesnean NALMA) of Utah (Hutchison, pers. obs.). The Uintan NALMA therefore represents a unique period of insight into the morphological variation and evolution near the end of the baenid radiation. Collection of various turtles, including baenids, have been one focus of larger project evaluating the vertebrate diversity and evolution from the middle Eocene sediments represented in the Uinta Basin, Utah. These specimens have been collected within the context of a high-resolution stratigraphic section that allows us to better understand how vertebrates responded to global climatic conditions surrounding a hypothermal event [12–16]. Changing climatic conditions and local drainage reorganization including the westward regression of Lake Uinta (a major hydrologic feature of the early Eocene) likely had a profound effect on the evolution of baenids as they are no longer present in the Paleogene record after this epoch [17–19]. Therefore, these late surviving Uintan baenids provide insight into their evolution and ecological adaptations just prior to the extinction of this previously abundant and specious clade.

Taxonomy within the genus *Baena* has been the subject of much debate [2, 6, 8]. Since its inception, the number of species included in *Baena* has varied from as many as ten [8] to as few as one, *B*. *arenosa* [2]. Currently, two contemporaneous baenid taxa are unequivocally recognized from the Uintan NALMA—*Baena arenosa* [20] and *Chisternon undatum* [21]. An additional Eocene species, “*Baena*” *affinis*, was recently resurrected by Joyce and Lyson [6], and is known primarily from partial shells from the Bridgerian NALMA (50.3–46.2 Ma). However, no specimens from the Uintan NALMA have been specifically attributed to *“B*.*” affinis*, as of yet. *Baena arenosa* differs from *“B*.*” affinis* on the basis of two primary traits: presence of prepleural scales, and a reduced number of inframarginal scales [6, 22]. All of the new Uinta material we ascribe to *B*. *arenosa* lack prepleurals. None of the newly discovered Uintan specimens described in this study preserve the inframarginal region with sufficient detail to assess the latter character. In addition, prepleural scales are also found in coeval Uintan *C*. *undatum* fossils, and the few specimens we observed with a prepleural scale also displayed a suite of characters that aligned them with *Chisternon*. Thus, we conservatively refer the new *Baena* specimens described herein to *B*. *arenosa*, rather than *“B*.*” affinis*.

Cessation of growth and co-ossification, such as in baenids, are rare among turtles. Most turtle species are considered to grow indeterminately, although the rate of growth progressively reduces with age [23–26]. The fusion of individual bony elements often makes baenid fossils less vulnerable to taphonomic damage than sympatric turtle taxa with unfused shell elements and crania, resulting in a disproportionately high number of baenid skulls in museum collections [26]. Co-ossification may also obscure taxonomically informative morphological features and render descriptions of individual bony elements ambiguous.

Associated cranial and shell material of baenid specimens are comparatively rare in the fossil record [6]. Despite the general over-representation of baenid crania in museum collections, Gilmore (1916) described the only previous baenid skull from the Uinta Basin. A slightly crushed and distorted, but relatively complete skull of *B*. *arenosa*, CM 2956, was recovered from the Uinta C horizon, Myton Member [2, 9]. Upon its discovery, CM 2956 was originally ascribed to “*Baena*? *sp*. *indet*.” due to the lack of associated diagnostic shell material [9], but Gaffney [2] later subsumed this within *B*. *arenosa*. The cranial cavity and foramina of CM 2956 are filled with hardened sediment; thus, the internal anatomy of the skull is not readily visualized. Gaffney [2] attributed most of the observed differences between CM 2956 and earlier Bridgerian *B*. *arenosa* skulls to distortion and crushing in the former; however, this assertion could not be confirmed due to the absence of other Uintan *B*. *arenosa* crania in the fossil record.

Here, we present a new specimen of *B*. *arenosa*, including a partial skull with associated vertebral, scapular, and shell elements. This skull is only the second baenid cranial specimen reported from the Uinta Basin, and one of the two youngest described skulls from the entire Baenidae clade, along with CM 2956. The internal surface of our new specimen is free from matrix, and the detailed morphology of the internal aspect of the basicranium and neurocranium is described for the first time in *B*. *arenosa* from the Uinta Basin. We also describe shells of several additional Uintan *B*. *arenosa* and *C*. *undatum* specimens.

## Materials and methods

New Uintan baenid material comprises 27 individual specimens, containing cranial, postcranial, carapacial and plastral elements, and is described in detail below (Table 1). New material for *Baena arenosa* includes a partial skull with several associated postcranial bones and various associated shell elements (UMNH VP 27535), described first, followed by a description of 21 additional new *Baena arenosa* shell specimens, including two subadult individuals. The *Chisternon undatum* material includes five new specimens, including one nearly complete, articulated plastron and carapace, three other partial adult *Chisternon* specimens, and one subadult partial carapace and plastron. Fossil material representing both genera were recovered from numerous localities in the Uinta Formation, Uinta Basin, Utah. Fossils were collected under a permit issued by the Bureau of Land Management (BLM) to KET. Specimens have been accessioned into the Vertebrate Paleontology collection of the Natural History Museum of Utah, Salt Lake City, UT. Museum accession numbers are listed in Table 1. Measurements were taken using Mitutoyo Hillson-Fitzgerald digital dental calipers [27] with an accuracy of 0.01 mm. Data for specimens held in museums were taken from the literature, museum databases, and by data taken from loaned museum specimens.

**Table 1:** Locality, meter level, and stratotype information for *Baena arenosa* and *Chisternon undatum* specimens described in the present study.

Specimen Number	Locality	Meter Level (m)	Strato-type	Taxon	Developmental Status		Element(s) present
UMNH VP 27085	WU-18	25	Ui2	*Baena arenosa*	Adult	Partial plastron	
UMNH VP 26729	WU-6	25	Ui2	*Chisternon undatum*	Adult	Carapace and plastral sections	
UMNH VP 27191	WU-8	57/60	Ui2	*Baena arenosa*	Adult	Nearly complete carapace and plastron	
UMNH VP 27192	WU-8	57/60	Ui2	*Baena arenosa*	Adult	Posterior plastron, partial carapace	
UFH 11739	WU-25	60	Ui2	*Baena arenosa*	Adult	Anterior carapace fragment	
UMNH VP 27535	WU-22	87	Ui2	*Baena arenosa*	Adult	Partial skull, postcranial and shell fragments	
UMNH VP 27604	WU-22	87	Ui2	*Baena arenosa*	Adult	Nearly complete carapace	
UMNH VP 27319	WU-22	87	Ui2	*Chisternon undatum*	Adult	Anterior carapace	
UMNH VP 27544	WU-22	87	Ui2	*Chisternon undatum*	Subadult	Carapace and plastral sections	
UMNH VP 27554	WU-22	87	Ui2	*Chisternon undatum*	Adult	Nearly complete carapace and plastron	
UMNH VP 27542	WU-83	87	Ui2	*Baena arenosa*	Adult	Nearly complete plastron, carapace fragments	
UMNH VP 27543	WU-83	87	Ui2	*Baena arenosa*	Adult	Anterior plastron	
UMNH VP 27652	WU-83	87	Ui2	*Chisternon undatum*	Adult	Anterior carapace, plastron fragments	
UMNH VP 27653	WU-83	87	Ui2	*Baena arenosa*	Adult	Anterior carapace	
UMNH VP 27540	WU-34	96	Ui2	*Baena arenosa*	Subadult	Posterior plastron, midline carapace	
UMNH VP 27541	WU-34	96	Ui2	*Baena arenosa*	Adult	Partial carapace, plastral fragments	
UMNH VP 27545	WU-34	96	Ui2	*Baena arenosa*	Adult	Partial plastron, carapace fragments	
UCMP 179283	WU-54	96	Ui2	*Baena arenosa*	Adult	Nearly complete carapace, plastron	
UMNH VP 27338	WU-2	104	Ui2	*Baena arenosa*	Adult	Partial plastron	
UMNH VP 27538	WU-2	104	Ui2	*Baena arenosa*	Adult	Nearly complete plastron	
UMNH VP 27547	WU-13	140	Ui3	*Baena arenosa*	Subadult	Carapace section	
UCMP 179495	WU-222	332	Ui3	*Baena arenosa*	Subadult	Partial carapace	
UCMP 179496	WU-222	332	Ui3	*Baena arenosa*	Subadult	Partial carapace and plastron	
UCMP 179520	WU-222	332	Ui3	*Baena arenosa*	Subadult	Partial carapace, plastron fragments	
UMNH VP 27537	WU-223	332	Ui3	*Baena arenosa*	Subadult	Partial plastron, midline carapace	
UMNH VP 27539	WU-223	332	Ui3	*Baena arenosa*	Adult	Anterior carapace	
UMNH VP 27546	WU-123	366	Ui3	*Baena arenosa*	Adult	Anterior plastron, carapace sections	

### Geological setting

The Uinta Formation (Uinta Basin), located in Utah, is a highly fossiliferous region consisting of over 265 productive fossil localities [13]. It is the type formation of the Uintan North American Land Mammal Age (NALMA) [11, 28]. Since 1994, researchers from Washington University and Midwestern University conducted fossil collection and stratigraphic work in the Uinta Basin [11, 13, 15, 29–32]. Although the collection of mammal taxa from these localities has been a greater historic priority, many turtle genera are also abundant.

**Fig 1:**
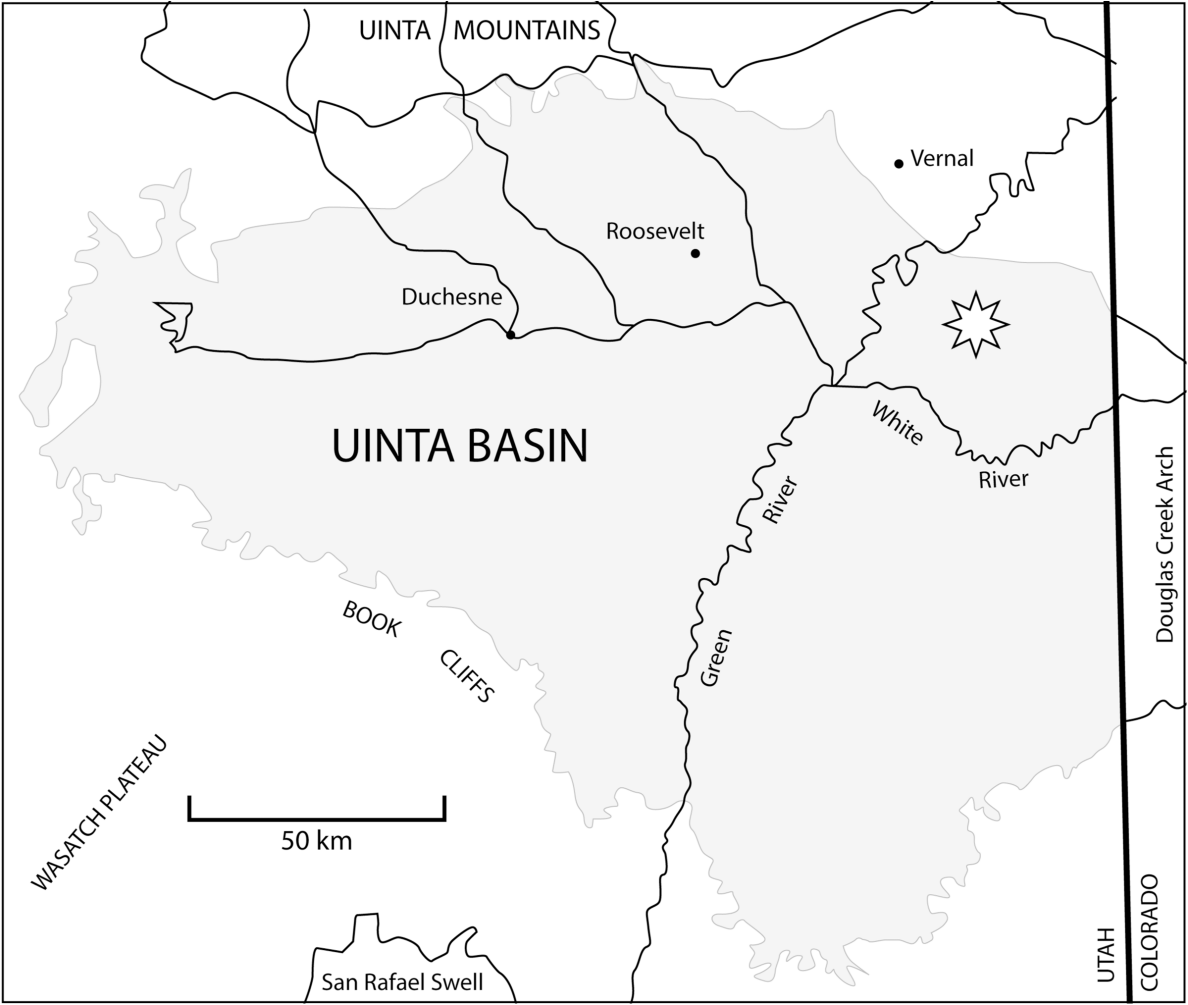
Map showing the geographic location of the Uinta Basin, Utah. The gray region demarcates the Tertiary deposits of the Uinta Basin (Utah) and Piceance Creek (Colorado). Star indicates the study area of Uintan NALMA localities from which the baenid specimens described herein were recovered, between the Green and White Rivers.

The study site is flanked by the Green River and White Rivers, between latitudes 40°00’ and 40°30’ north and longitudes 109°00’ and 109°45’ west [13] (Fig 1). The localities reported here are tied to a section published by Townsend et al. [13] that extends 366 m through the older lithostratigraphic unit Uinta B (0–137 m) to the younger Uinta C (140–366 m), and at 366 m is the first conformable contact between the Uinta Formation and Duchesne River Formation [11, 13, 33–34]. Gunnel and colleagues [35] divided the Uintan NALMA into four biochronological zones—Ui1a, Ui1b, Ui2, and Ui3—on the basis of mammalian biostratigraphy from the Bridger, Uinta, and Washakie Formations. The stratotypes for the biochrons Ui2 and Ui3 occur in the immediate area of the Uinta Basin, and our localities from where the turtles were recovered are within these stratotype sections or can be stratigraphically correlated with them [15, 35].

### Abbreviations

The following institutional abbreviations will be used: CM, Carnegie Museum of Natural History; MCZ, Museum of Comparative Zoology, Harvard University; NHMU/UMNH VP, Natural History Museum of Utah; UCMP, University of California Museum of Paleontology; UFH, Utah Field House of Natural History State Park Museum; YPM-VP, Yale Peabody Museum- Vertebrate Paleontology. The following anatomical abbreviations will be used: M, muscle.

### Phylogenetic analyses

In order to determine whether the new Uintan baenid specimens affected the current understanding of phylogenetic relationships within the Baenidae, we conducted a phylogenetic analysis. We used as our starting point, a recent analysis of baenid phylogeny [36] which included a compilation of 69 characters from Lyson and Joyce [5, 37–39], Larson et *al*. [40], and novel characters [36]. We assessed all of these characters on the new Uintan specimens. We then amended the previously proposed codification for *B*. *arenosa* and *C*. *undatum* accordingly (S1 File). All characters were equally weighted. Following Lyson et al. [36], characters 1, 10, 12, 15, 21, 22, 24, 30, 37, 41, 44, 56, and 62 were treated as ordered, while all others were unordered in the analysis. See Lyson et al. [36] for further details. A maximum parsimony analysis was conducted in Tree Analysis using New Technology (TNT) v1.5 [41], using a traditional heuristic search with tree bisection reconnection (TBR) swapping algorithm. Tree length, consistency index (CI), and retention index (RI) were recorded.

## Results

### Systematic paleontology

TESTUDINES Batsch, 1788 [42]

PARACRYPTODIRA Gaffney, 1975 [43]

BAENOIDEA Williams, 1950 [44]

BAENIDAE Cope, 1882 [1]

BAENODDA Gaffney and Meylan, 1988 [45]

*BAENA ARENOSA* Leidy, 1870 [20]

**Fig 2:**
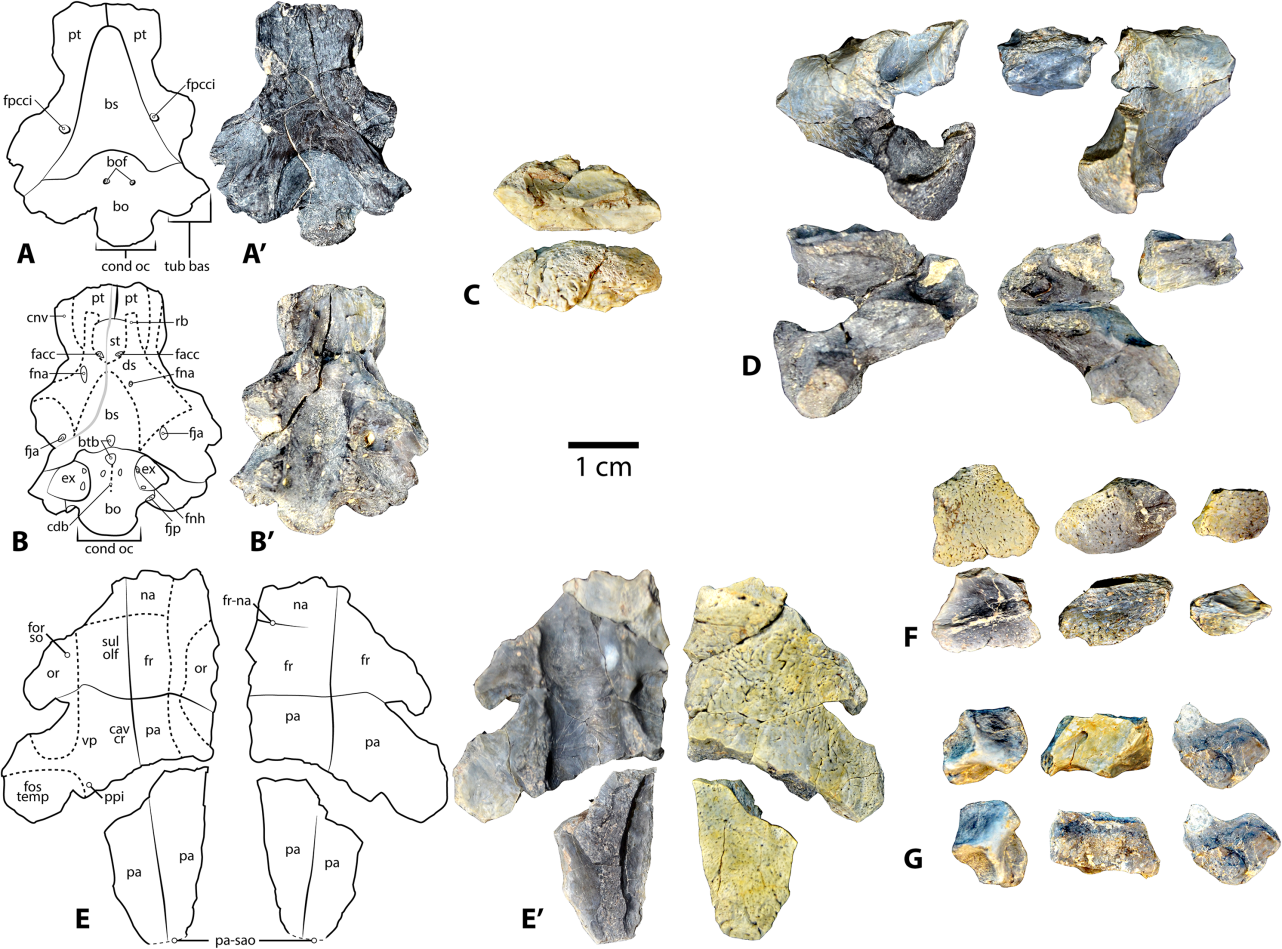
Cranial elements from *Baena arenosa* specimen UMNH VP 27535 from the Uinta Basin, Uinta Formation, Utah. (A-A') Ventral view of basicranium. (B-B') Dorsal view of basciranium. (C) Right squamosal: Medial view (above), lateral view (below). (D) Left and right quadrates (on left and right, respectively): Anterior view (above), posterior view (below). (E-E') Neurocranium: Ventral view (left), dorsal view (right). (F) Maxillary fragments: Lateral view (above), medial view (below). (G) Lower jaw fragments: Articular (left), prearticular (middle), coronoid (right); Internal view (above), external view (below). Abbreviations: bo = basioccipital; bof = basioccipital foramen; bs = basisphenoid; btb = basis tuberculi basalis; cav cr = cavum cranii; cdb = crista dorsalis basioccipitalis; cnv = canali nervi vidiani; cond oc = condylus occipitalis; ds = dorsum sellae; ex = exoccipital; facc = foramina anterius canalis carotici interni; fja = foramen jugulare anterius; jjp = foramen jugulare posterius; fna = foramen nervi abducentis; fnh = foramen nervi hypoglossi; for so = foramen supraorbitale; fos temp = fossa temporalis; fpcci = foramen posterius canalis carotici interni; fr = frontal; fr-na = fronto-nasal suture; na = nasal; or = orbit; pa = parietal; pa-sao = parietal-supraoccipital suture; ppi = processus parietalis inferior; pt = pterygoid; rb = rostrum basisphenoidale; st = sella turcica; sul olf = sulcus olfactorius; tub bas = tuberculum basioccipitale; vp = vertical plate of parietal.

**Fig 3:**
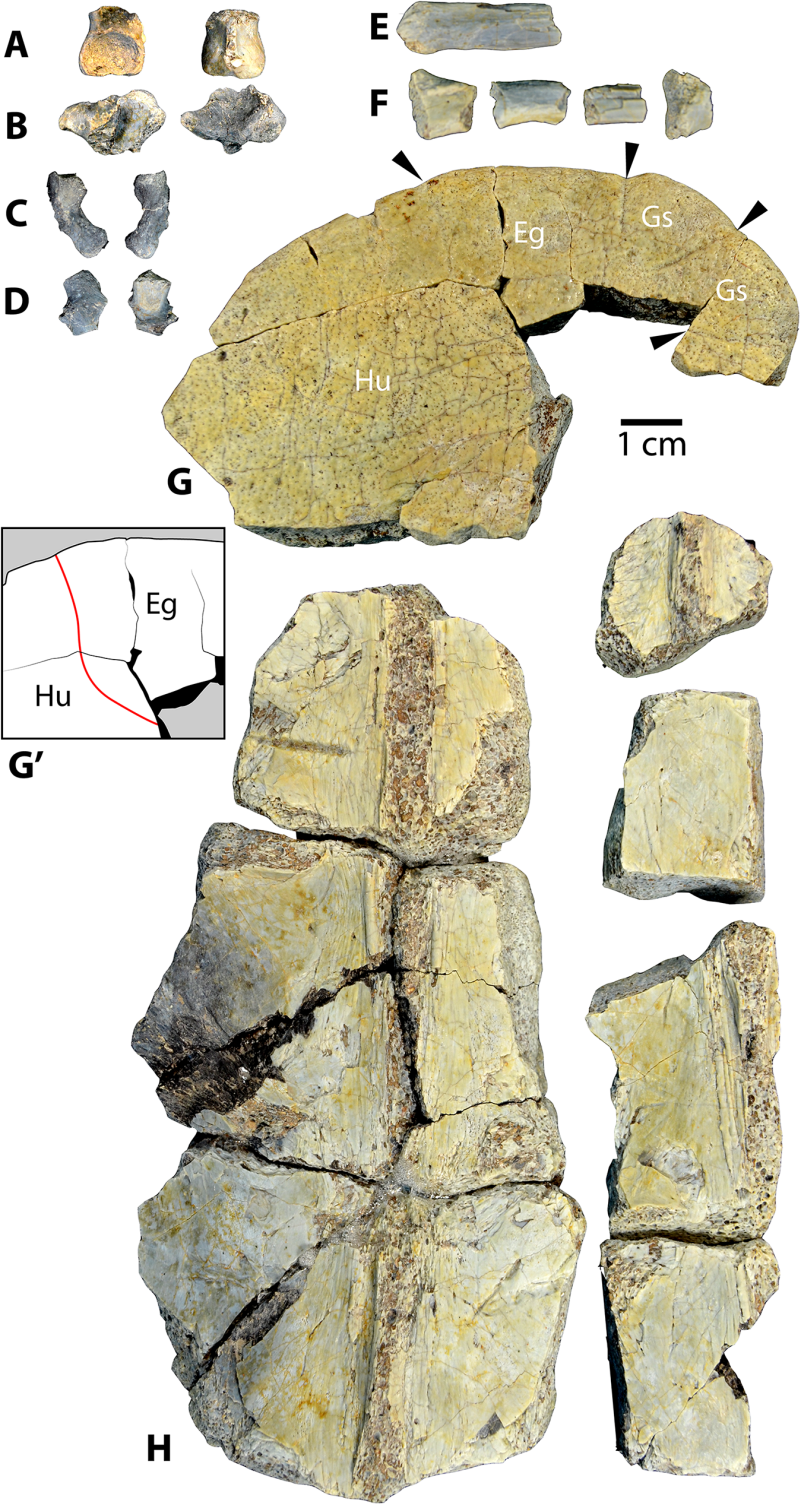
Postcranial elements from Uintan *Baena arenosa* specimen UMNH VP 27535. (A) Vertebral centrum: Posterior view (left), anterior view (right). (B) Vertebral centrum with transverse process: Posterior view (left), anterior view (right); (C-D) Cervical ribs: Caudal surface (left), cranial surface (right). (E) Scapular blade: Rostral surface. (F) Long bone fragments. (G) Anterior plastral section: Dorsal view. (G') Schematic depiction of epiplastron illustrating sigmoidal shaped humeral-extragular sulcus (in red). (H) Neural elements: Ventral view. Black arrows indicate positions of plastral sulci. Abbreviations: Eg = Extragular scale; Gs = Gular scale; Hu = Humeral scale.

Referred specimens: Specimen UMNH VP 27535 includes a fragmentary skull of *Baena arenosa* (Fig 2A–2F), as well as numerous associated incomplete shell elements and several limb bone fragments (Fig 3). The cranial regions preserved include the basicranium, a neurocranial fragment, both otic regions, several isolated facial pieces, and a few fragments of the lower jaw. The shell fragments consist of a partial neural row, epiplastron, and several partial costals, peripherals, and bridge elements. A small number of isolated postcranial bones include the distal end of a scapular blade and portions of a few cervical vertebrae. Dimensions of individual cranial elements and features are listed in Supplementary Table 1.

### Description and comparison

#### Basicranium

The basicranium of specimen UMNH VP 27535 includes a complete basioccipital and basisphenoid, the caudoventral portions of the left and right pterygoids, and portions of the exoccipitals (Fig 2A, 2A', 2B and 2B'). The basicranium is smaller in size than that of CM 2956, suggesting a smaller overall cranial (and likely body) size. While the left pterygoid retains a suture between it and the left margins of the basioccipital and basisphenoid, the other cranial bones and much of the carapace have fused to the point that their sutures have been entirely obliterated (Fig 2A and 2B).

The basioccipital contacts the pterygoids posterolaterally. The ventral surface of the basioccipital contains a deep (2.7 mm), rounded depression in the midline (12.0 mm wide x 8.4 mm long), bounded laterally on either side by prominent tuberculum basioccipitale (Fig 2A and 2A'. The depression is more pronounced than in CM 2956 and MCZ 4072, with a distinctly demarcated margin. Several small foramina are present within the midsagittal region of the depression (Fig 2A). The tuberculum basioccipitale flare out laterally, and their tips are slightly indented. The basisphenoid is relatively short, and the ventral side is triangular in shape and flattened (Fig 2A). The basisphenoid-pterygoid suture is broad. A deep groove runs parallel to the left margin of the basisphenoid approximately 1 mm from its suture with the left pterygoid, and 6.2 mm in length. Ventral to the basisphenoid rostrum, the two pterygoids contact each other extensively (Fig 2A). The two foramina posterius canalis carotici interni are large (1.3 mm diameter), rounded, and contained entirely within the pterygoid with no basisphenoid contact (Fig 2A and 2A').

The condylus occipitalis in UMNH VP 27535 is crescent-shaped, and concave dorsally. Its neck is distinctly constricted at the base, and widens laterally as it extends caudally, creating a knob-like condylus (Fig 2B and 2B'). The pit on the condylus occipitalis attachment of the ligamentum apicis dentis [46–49] is shallow and subtle, as in CM 2956. The ventral margin of the foramen magnum is preserved, and is apparently mediolaterally wider than that of CM 2956; however, the right lateral margin and dorsal margin of the foramen are not present in UMNH VP 27535, so its diameter is not directly measureable. Based on the distance between the left lateral margin and the midline, we estimate its width at approximately 7.4 mm. Portions of both exoccipitals are present, but the left side is more complete and includes most of both the dorsolateral and ventral processes (Fig 2B and 2B').

Only the ventral process of the right exoccipital is preserved (Fig 2B and 2B'). The paired foramina nervi hypoglossi are present bilaterally, but are better preserved on the left side. The internal openings into the canalis hypoglossi from inside the foramen magnum are round and distinct, with the caudal-most foramen being slightly larger than the more cranial one (Fig 2B). The paired external foramina nervi hypoglossi on the lateral side of the left exoccipital are positioned closer to each other than they are on the right, with the larger caudal foramen occupying a slightly more dorsal position (caudal foramen: 0.9 mm; ventral foramen: 0.8 mm) than the smaller foramen. The dorsolateral portion of the right exoccipital is broken, exposing the more caudally positioned of the two canalis hypoglossi. The canal is 4.3 mm in length and 0.7 mm in diameter, and takes a slightly curved course through the exoccipital bone to open laterally. A small oval-shaped foramen is present, slightly caudal to the crista dorsalis basioccipitalis, just to the right of the midline (Fig 2B and 2B'). We interpret this to be a third, more medially positioned foramen nervi hypoglossi. This supernumerary foramen is not atypical in testudinoids [46–47]. The foramina jugulare posterius are preserved bilaterally, and are large and oval-shaped (Fig 2B). The left side is slightly distorted, but the right side appears to have maintained its origin dimensions (3.3 mm wide x 2.3 mm high). The foramina jugulare posterius face caudally, and sit in a depression between the tuberculum basioccipitale and the large, projecting condylus occipitalis, the latter of which creates a ridge overhanging the superior margin of the foramina jugulare posterius (Fig 2B and 2B').

The crista dorsalis basioccipitalis is subtle and almost insignificant, leading cranially to a small, circular basis tuberculi basalis (0.6 mm) (Fig 2B and 2B'). A small right foramen nervi abducentis (0.5 mm diameter) is present approximately halfway up the lateral side of the caudal margin of the dorsum sellae (Fig 2B). The caudal surface of the dorsum sellae ascends gradually from the horizontal portion of the basisphenoid, leading up to a flattened peak (Fig 2B and 2B'). Its anterior surface is flat and declines steeply, almost directly vertically with minimal overhang. The sella turcica is round, approximately 4.5 mm in diameter and 2 mm deep. The paired foramina anterius canalis carotici interni are rounded and approximately 0.8 mm in diameter (Fig 2B and 2B'). The rostrum basisphenoidale project anteriorly 5.4 mm beyond the sella turcica (Fig 2B and 2B'). The right side is partially weathered, revealing part of the canalis caroticus lateralis coursing within the rostrum. The large canali nervi vidiani are open dorsally, exposing portions of the vidian canals bilaterally (Fig 2B and 2B'). There is a small, medially oriented foramen in the medial wall of each canal, which likely represents the opening of a transverse communication between the vidian canal and the canalis caroticus lateralis, as has been described in *Baena arenosa* (YPM-VP 3941) and *Chisternon undatum* (YPM-VP 3930) basicrania [48].

#### Otic region

The left and right quadrates are in good condition, and a portion of the right squamosal is present (Fig 2C and 2D). They are broad, thick bones, and from a posterior view, they appear “C-shaped” as in other baenids [48] (Fig 2D). The contribution of the quadrate to the fossa temporalis superior suggests a minimal degree of temporal emargination, comparable to that of other *B*. *arenosa* specimens. The processus trochlearis oticum of UMNH VP 27535 is wide but unpronounced, consisting primarily of a subtle, sloping projection into the temporal fossa (Fig 2D). This process is generally unremarkable in baenids [8]. In life, this process serves as the attachment point for the tendons of the lower jaw adductor muscles [46], which were presumably less developed in baenids than most other cryptodires.

The processus articularis is quite short, noticeably less projecting than in CM 2956. The articular surface of the condylus mandibularis is shallow (1.5 mm), and its medial and lateral margins do not extend far ventrally (Fig 2D). In most Testudines, this joint surface consists of two distinct articular facets separated by a groove [46]. However, in UMNH VP 27535 no facets are visible and the entire articular surface appears worn (Fig 2D). It bears several nutrient foramina, and roughening on the surface, which may indicate osteoarthritic changes in the jaw joint or taphonomic alterations. A groove runs along the medial side of the processus articularis, and onto the posterior side, terminating in a small pit. This feature occurs bilaterally in UMNH VP 27535, and is also observed on MCZ 4072, although the groove on the latter is less pronounced. The foramen chorda tympani inferius is slightly ovate. The foramen chorda tympani superius is not preserved on either side.

The ventral margin of the otic capsule is preserved, and suggests a broad, rounded opening leading into the cavum tympani (Fig 2D). The projection below the external otic opening is elongated and triangular. Medial to the otic margin, the cavum tympani dips slightly ventrally, and then opens up into an expanded, smooth-walled cavity (Fig 2D). The dorsal margin of the left incisura columellae auris is preserved, indicating a broad, rounded foramen. It is open caudally, as described in other baenids [6]. Just distal to the incisura columellae auris, the cavum acustico-jugulare expands medially. The canalis cavernosus and aditus canalis stapedio-temporalis diverge further medially. The canalis cavernosus consists of a deep, straight furrow, demarcated by sharp margins. The broad aditus canalis stapedio-temporalis constricts to an extremely round canalis stapedio-temporalis. The medial third of the foramen stapedio-temporale is preserved, and suggests a circular shape of the foramen. The lateral margin of this foramen is not preserved.

The suture for articulation with the squamosal is elliptical, and its surface is extremely smooth with the appearance of a facet surface (Fig 2D). The antrum postoticum is deep and its surface smooth. The suture with the posterior portion of the pterygoid is roughly rectangular, and appears to lack the typical medial dorso-ventral expansion that gives the quadrate-pterygoid suture its triangular shape in many cryptodires [46]. The processus epipterygoideus is broad and squat, and does not extend considerably beyond the medial boundary of the pterygoid suture. The posterolateral portion of the right squamosal is present, including the lateral margin of the antrum postoticum. Its ventral surface contains the lateral part of a smooth and rather shallow fossa for attachment of the M. depressor mandibulae [50–53], while its external surface is extremely crenulated (Fig 2D). Posteriorly, it appears to taper to a significant projection, comparable to that of CM 2956.

#### Neurocranium

Specimen UMNH VP 27535 contains a moderately-sized (25 mm long x 27 mm wide) midline neurocranial fragment, which includes most of the left and right frontal bones, the anterolateral portion of the right parietal, a small section of the anterior-most part of the left parietal, and the left nasal (Fig 2E and 2E'). It incorporates small portions of the left and right orbital margins, as well as the left side of the superior margin of the nasal aperture. Several other fragmentary, relatively undiagnostic cranial vault fragments are also present.

The dorsal skull roof of UMNH VP 27535 is thick (3.6 mm at the midline of the parietals), as Joyce and Lyson [6] described as characterizing *Baena arenosa*. The frontal bones are broad and flat, and roughly rectangular in shape with a subtly convex shape (Fig 2E and 2E'). They contact the nasals anteriorly, parietals posteriorly, and each other medially. The adjacent sections of maxilla, prefrontal, and postorbital are absent, so it cannot be determined whether the frontals contact these bones in this specimen. The left frontal includes a moderately-sized suture for articulation with the maxilla, which is straight and transverse (Fig 2E). Gaffney [48] suggested that a well-developed fronto-maxillary contact is a synapomorphy of the Baenodda clade. The right frontal is more complete than the left, and contains more of the dorsal margin of the fossa orbitalis. While it is not possible to estimate the shape of the entire orbit, the contour of the dorsal margin suggests a rather circular orbit. In the left frontal, only 4 mm of the orbital margin immediately posterior to the fronto-nasal suture is preserved. Based on these limited sections, the orbits appear to be facing relatively laterally, a condition found in most baenid genera, except *Arvinachelys*, *Cedrobaena*, *Eubaena*, *Gamerobaena*, and *Palatobaena*, in which the orbits face dorsally [5, 36, 49]. A projecting posterolateral process is visible on the right side.

The dorsal surface of the parietals is broad and quite flat (Fig 2E and 2E'). The suture between the left and right parietals is fused but still visible, as is the fronto-parietal suture on either side, the latter of which is relatively straight. The parietals appear relatively large; however, the degree of their posterolateral extension cannot be determined due to breakage. A second isolated midline neurocranial fragment includes the posteriormost portions of the left and right parietals (Fig 2E and 2E'). The dorsal surface of this fragment is extremely smooth, with no indication of a crista supraoccipitalis that extends onto the parietals (Fig 2E and 2E'). This crest, when present, is typically small in baenids [46]. The suture for articulation with the supraoccipital is straight and short (4.5 mm) (Fig 2E). On the right side, the original posterolateral border of the parietal is retained, and is oriented at approximately 110° angle to the parieto-supraoccipital suture, suggesting that the posterior dorsal vault tapers to a midline projection. While the entire parietal bone is not present on either side, the combined length of the parietal fragments (28.5 mm) is still greater than twice as long as the frontal bones (12.3 mm), indicating that the parietals are substantially longer than the frontals in this specimen.

The ventral surface of the frontals consists of a single median sulcus olfactorius (Fig 2E and 2E'), which houses the olfactory nerve (CN I) [46]. Further caudally, the ventral portions of the parietals compose the anterior part of the cavum cranii. A faint transverse line separates the cavum cranii from the sulcus olfactorius. The vertical plate of the parietals separates the orbits from the cavum cranii (Fig 2E and 2E'). The dorsal part of the internal surface of the orbital margin suggests a large, circular orbit (Fig 2E). A round foramen supraorbitale is present on the right side (Fig 2E and 2E'). A small portion of the processus parietalis inferioris is present on the right, demarcating the anterior margin of the right fossa temporalis (Fig 2E and 2E'). The left frontal is fused with the vertical plate of the prefrontal, a small fragment of which is present in this specimen.

#### Facial skeleton

Of the facial skeleton, the anteromedial aspect of the right maxilla, two fragments of the left maxilla, and portions of the nasal bones are present in UMNH VP 27535 (Fig 2F). The maxilla includes the lateral margin of the aperture narium externa, and small sections of the anteroventral margin of the orbit, horizontal plate, and triturating surface. A single dorsal suture, apparently for articulation with the right frontal is also present, and is quite straight, horizontally oriented, and smooth. There is no discernible separate suture for articulation with the nasal.

Due to absence of the dorsal margin of the maxilla, the contribution of this bone to the anteroventral orbital margin is quite small (approximately 6 mm), but rounded. The internal surface of this contribution is recessed with a deep furrow (Fig 2F). The aperture narium externa appears to be quite rounded laterally (Fig 2F), as in CM2956 and MCZ 4072. The internal wall of the fossa nasalis contains a sloping fossa. Posterior to the fossa, the foramen alveolare superius is present, and is large and rounded as in CM 2956.

The horizontal portion of the maxilla attaches perpendicularly to the vertical plate. While only the lateral-most portion of the processus palatinis is preserved, it is still possible to estimate its lateral thickness at approximately 1.5 mm. Ventral to the processus palatinis, a series of numerous, tiny nutrient foramina populate the internal surface of the alveolar process. The alveolar process descends to terminate in a relatively thin and jagged triturating surface (Fig 2F). The labial ridge is noticeably thinner and its margin less even than in MCZ 4072 and CM 2956. The processus alveolus descends almost immediately vertically, without any medial curvature or lipping, such as in CM 2956 and MCZ 4072. It is possible that some damage has occurred to the margin of the triturating surface in this specimen, causing it to truncate prematurely. The lingual ridge is not preserved.

On the left side, the external surface of the maxilla is rugose, likely indicating the attachment of the horny rhamphotheca, and becomes increasingly crenulated ventrally (Fig 2F). A portion of the triturating surface is preserved. The labial ridge descends steeply; however, its dorsal-most projection is missing, so its height cannot be assessed. A shallow trough between the ridges is present (Fig 2F), as described in *B*. *arenosa* and *C*. *undatum* [48]. However, the labial ridge itself is not preserved. An additional section of crenulated facial bone, which may represent a section of jugal, is present and includes a portion of the orbital margin. The orbit appears to be comparatively small as in other *B*. *arenosa* specimens [2, 48], although its precise dimensions are not directly measurable. The contribution to the orbital margin by the jugal is quite extensive, a trait which Hay [8] described as characteristic of *B*. *arenosa* and *C*. *undatum*.

The nasals are small, and contact the frontal posteriorly and their antimere medially. As is typical in *Baena*, the midsagittal suture between the two nasals is completely obliterated. An apparent, straight suture is visible between the nasal and frontal on the left side, suggesting that these elements are separate bones in this specimen. The superior margin of the aperture narium externa is preserved on the left side, and it angles slightly dorsally from the midline creating a subtle “heart-shaped” appearance of the superior part of the aperture, similar but less pronounced than that of CM 2956. The anteriormost edge of the nasals are thickened and rugose with a midline depression. They converge anteriorly into a small midline projection, similar to the juvenile *Baena arenosa* specimen MCZ 4072. On the ventral surface of the nasals within the fossa nasalis, a delicate median ridge separates two parasagittal fossae, which Gaffney [46] suggested to be an extension of the sulcus olfactorius, and through which the olfactory bulbs of CN I course. A small round foramen is immediately posterior to the termination of the median ridge.

#### Lower jaw

Several fragments of lower jaw elements are also present in specimen UMNH VP 27535, including portions of the articular, coronoid, and prearticular (Fig 2G). The superior tip of the left coronoid is present. It includes a well-developed processus coronoideus, which is long and flattened (Fig 2G). Its lateral surface preserves a pronounced ridge, likely for the attachment of M. adductor mandibulae externus [46], and large rounded foramen dentofaciale majus [50, 52]. The anterolateral margin of the fossa meckelii is present, but it is not possible to estimate the size of the fossa.

Most of the left articular is present (Fig 2G). The posterior aspect is dominated by a large contribution to the area articularis mandibularis for articulation with the condylus mandibularis on the quadrate. The fossa is shallow and ovoid. The posterior process for attachment of the M. depressor mandibulae [46] is moderately sized and hooks slightly medially (Fig 2G). Gaffney [54] noted that this process is small in *Baena*, *Chisternon*, and *Plesiobaena*. Despite its small size in UMNH VP 27535, this process contains prominent ridges on either side. The foramen posterius chorda tympani is almost imperceptible. Anterior to the fossa, the articular widens into a broad, vertically oriented plate. A prominent lateral ridge connects the vertical plate to the posterior projection, creating a deep lateral fossa. A large foramen is also present on the medial surface. A fragment of right prearticular contains the foramen intermandibularis caudalis (Fig 2G). The foramen is not entirely surrounded by bone, such that it is open postero-dorsally (1.3 mm tall). In life, this foramen transmits a branch of the mandibular nerve (CN V_3_) [46]. It is typically located on the suture between the prearticular and angular; however, in UMNH VP 27535, there is no evidence of a suture, and the foramen appears to be positioned more dorsally than is typical.

#### Limb elements

Specimen UMNH VP 27535 contains a few isolated postcranial fragments (Fig 3A–3H). The distal portion of a scapular blade is present, suggesting an elongate, rod-like scapula (Fig 3E; Table 2). The caudal surface is round and smooth, while the rostral surface is slightly flattened. A prominent ridge courses obliquely along the rostral surface, indicating a well-developed levator scapulae-rhomboid complex (Fig 3E). The distal end expands slightly into a small knob-like termination with roughened depressions.

**Table 2:** Dimensions of postcranial bone elements in *Baena* specimen UMNH VP 27535.

Postcranial bone element	Dimensions
Scapular blade	7.3 mm dorsoventral width x 5.8 mm cranio-caudal width
Centrum of larger cervical vertebra	10.3mm wide x 8.4 mm long
Base of vertebral foramen in larger cervical vertebra	8.9 mm wide x 5.1 mm wide
Centrum of smaller cervical vertebra	5.8 mm long
Base of vertebral foramen in smaller cervical vertebra (est.)	5.7 mm wide
Transverse process (cervical vertebra)	6.3 mm long

Two fragments of cervical vertebrae of unknown position are present, a large, isolated centrum and a smaller centrum with a transverse process and prezygopophysis (Fig 3A and 3B; Table 2). On the large centrum, the depression on the articular surface is deeply concave, suggesting a cervical spine that articulated intimately (Fig 3A). It is an asymmetrical oval shape, mediolaterally wider than it is dorsoventrally long, resembling the anterior part of C8 in *C*. *undatum* specimen AMNH 5904 [8]. The proximal bases of the pedicles are widely spread, signifying a broad vertebral canal. The smaller centrum is more triangular in shape and appears convex, suggesting that UMNH VP 27535 may be partly opisthocoelous (Fig 3B). The transverse process is robust, with rugose muscle attachment markings. It angles faintly posteriorly from the centrum, and then curves slightly postero-ventrally at its lateral-most tip. The vertebral foramen is larger in this element, and its ventral surface is quite flattened and smooth. The prezygopophysis is small, and its articular facet saddle-shaped. The bases of ventral keels are present on both centra, and moderately developed (Fig 3A and 3B); however, they are worn, and it is not possible to definitively assess their length.

Portions of two cervical ribs are present, one mostly complete rib and one isolated medial end (Fig 3C and 3D). Cervical ribs in UMNH VP 27535 are short, broad, and curved (Fig 3C and 3D). The cranial surface is rounded, while the caudal surface is flattened, and slightly concave. The proximal end is dominated by three, large flattened facets: one oriented medially, one posteriorly, and one dorsally. The articular facet on the head for attachment to the vertebral centrum is oval and concave. The tubercle of the rib consists of a large, teardrop-shaped facet, tapering distally, for articulation with the transverse process (Fig 3C and 3D). In the complete cervical rib (Fig 3C), the body does not taper distally; rather, the distal end widens out from the body of the rib. The distal end contains two small, ventrally positioned tubercles. Both cervical ribs in UMNH VP 27535 derive from the left side of the body, indicating the presence of cervical ribs at multiple vertebral levels, not just the atlas.

There are also a few undiagnostic remnants of long bone diaphyses associated with this specimen (Fig 3F). A small number of isolated shell fragments are also present, and are described in detail below in conjunction with the other *Baena arenosa* shell specimens.

#### Shells

**Fig 4:**
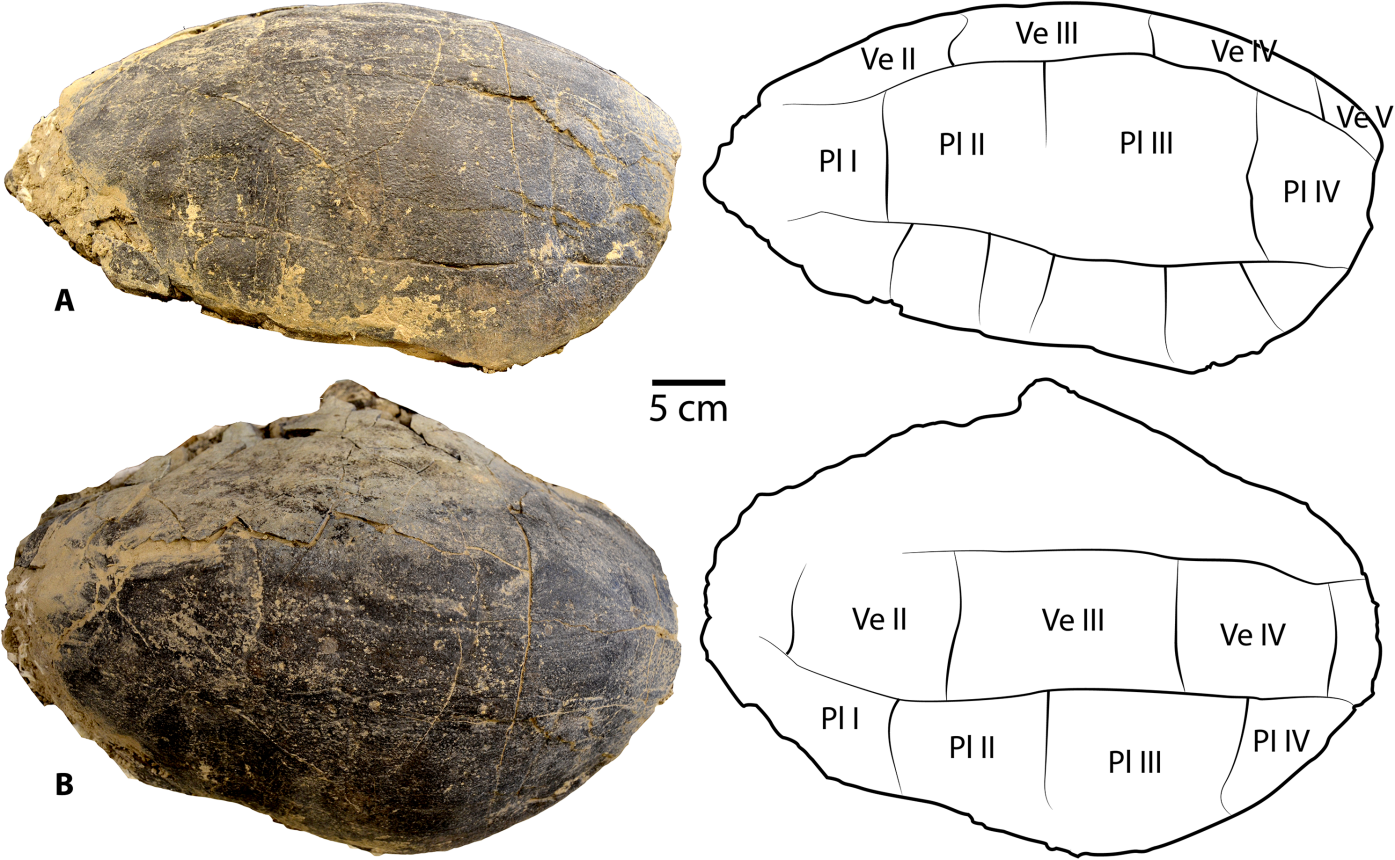
*Baena arenosa* carapace specimen from the Uinta Basin, UMNH VP 27604. (A) Right lateral view of the carapace of UMNH VP 27604. (B) Superior view of the carapace of UMNH VP 27604. Note the original curvature of the dome of UMNH VP 27604, one of the most complete *B*. *arenosa* carapace specimens from the Uinta Basin. Abbreviations: Pl = pleural scale; Ve = vertebral scale.

**Fig 5:**
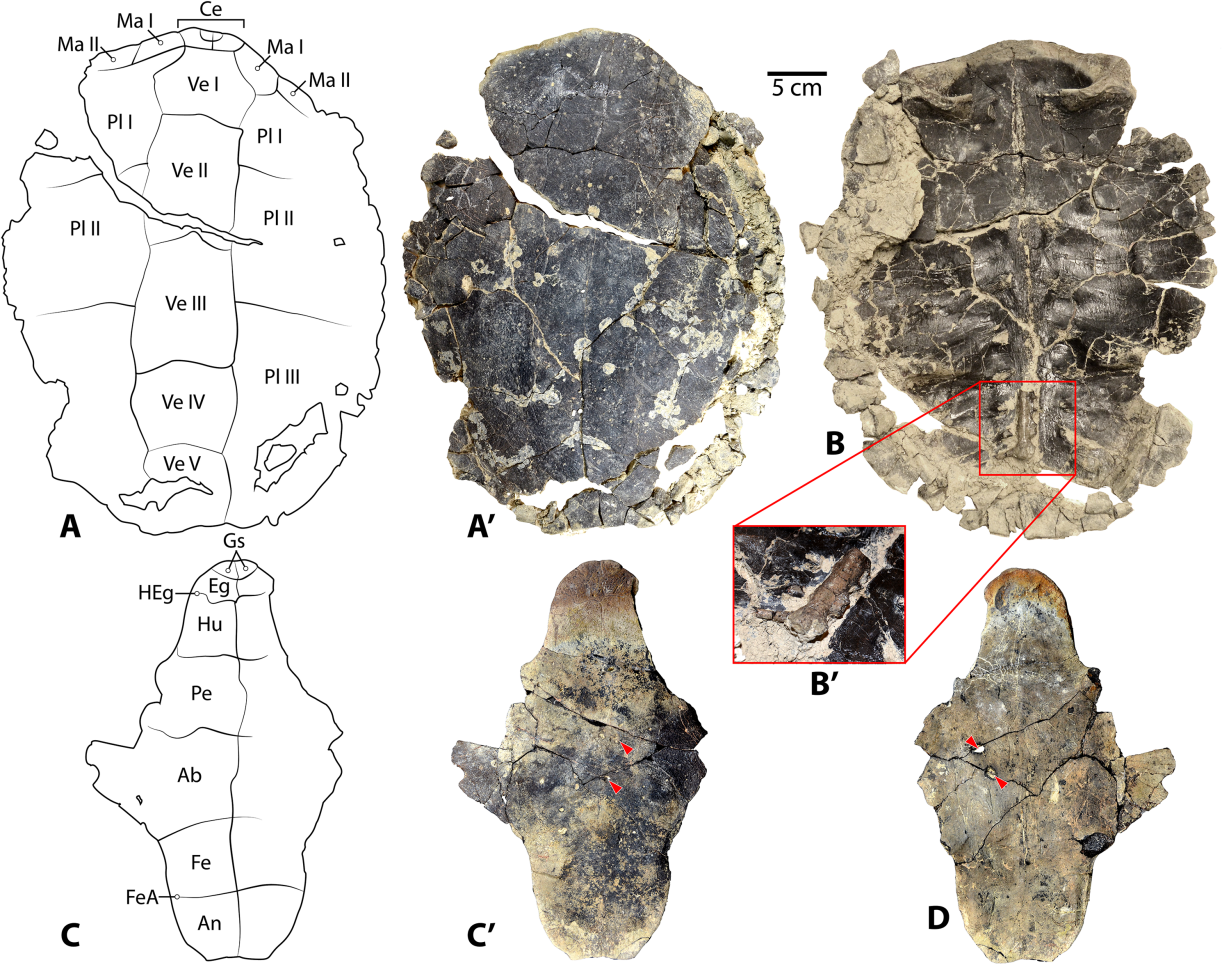
Carapace and plastron of Uintan *Baena arenosa* specimen UMNH VP 27191. (A-A') Dorsal view of carapace. (B) Ventral view of carapace. (B') Increased magnification of vertebral elements affixed to internal surface of carapace. (C-C') Ventral view of plastron. (D) Dorsal view of plastron. Red arrows indicate puncture pits consistent with carnivore bite marks. Abbreviations: Ab = abdominal scale; An = anal scale; Ce = cervical scale; Eg = extragular scale; Fe = femoral scale; FeA = femoral-anal sulcus; Gs = gular scale; Hu = humeral scale; HEg = humeral-extragular sulcus; Ma = marginal scale; Pe = pectoral scale; Pl = pleural scale; Ve = vertebral scale.

**Fig 6:**
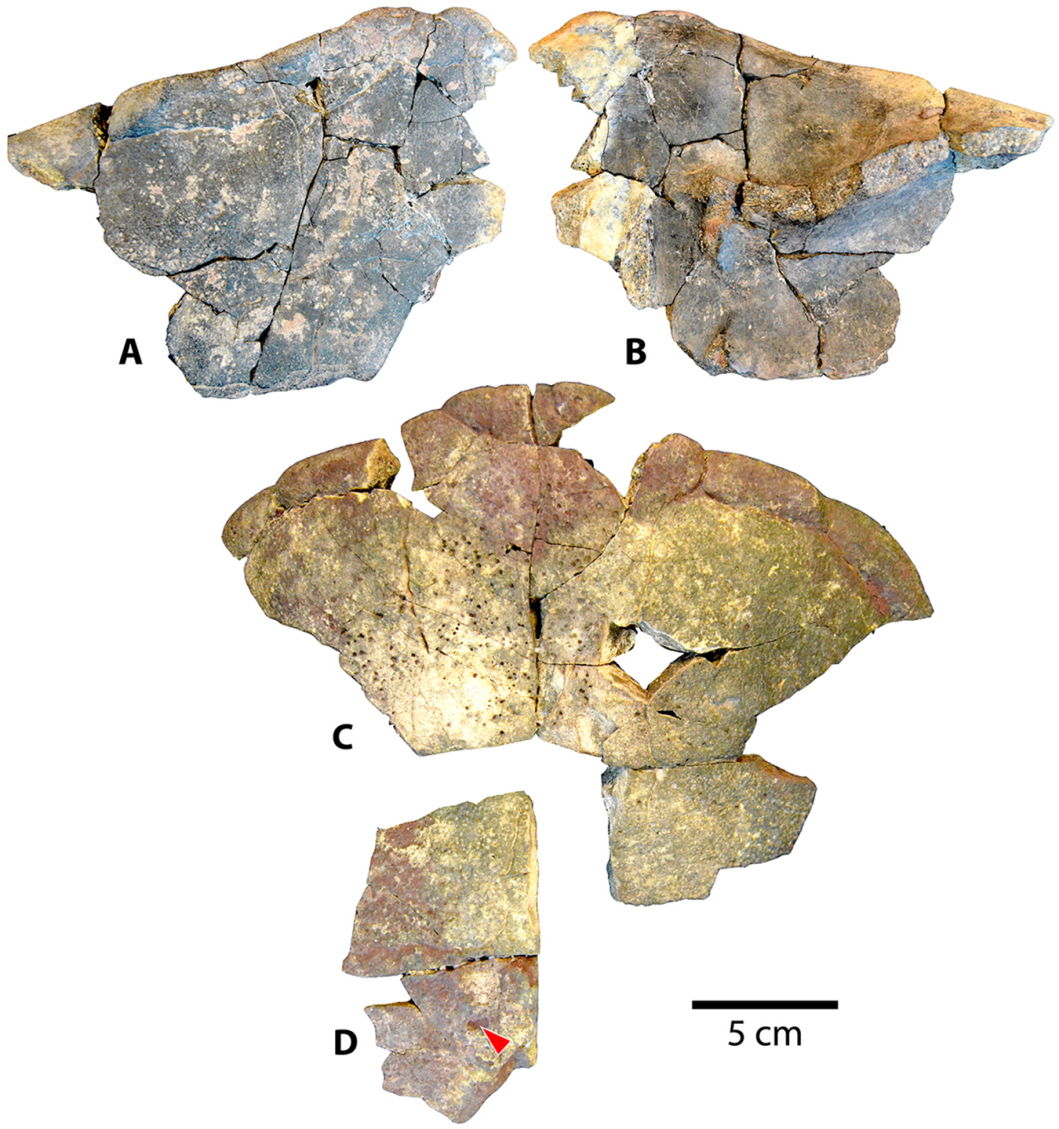
Two anterior carapace specimens of Uintan *Baena arenosa*. (A) Dorsal view of UMNH VP 27653 carapace. (B) Ventral view of UMNH VP 27653 carapace. (C) Anterior carapace of UMNH VP 27539 (dorsal view). (D) Mid-carapace section of UMNH VP 27539 with pitting (dorsal view). Red arrow indicates puncture pit consistent with carnivore tooth mark.

**Fig 7:**
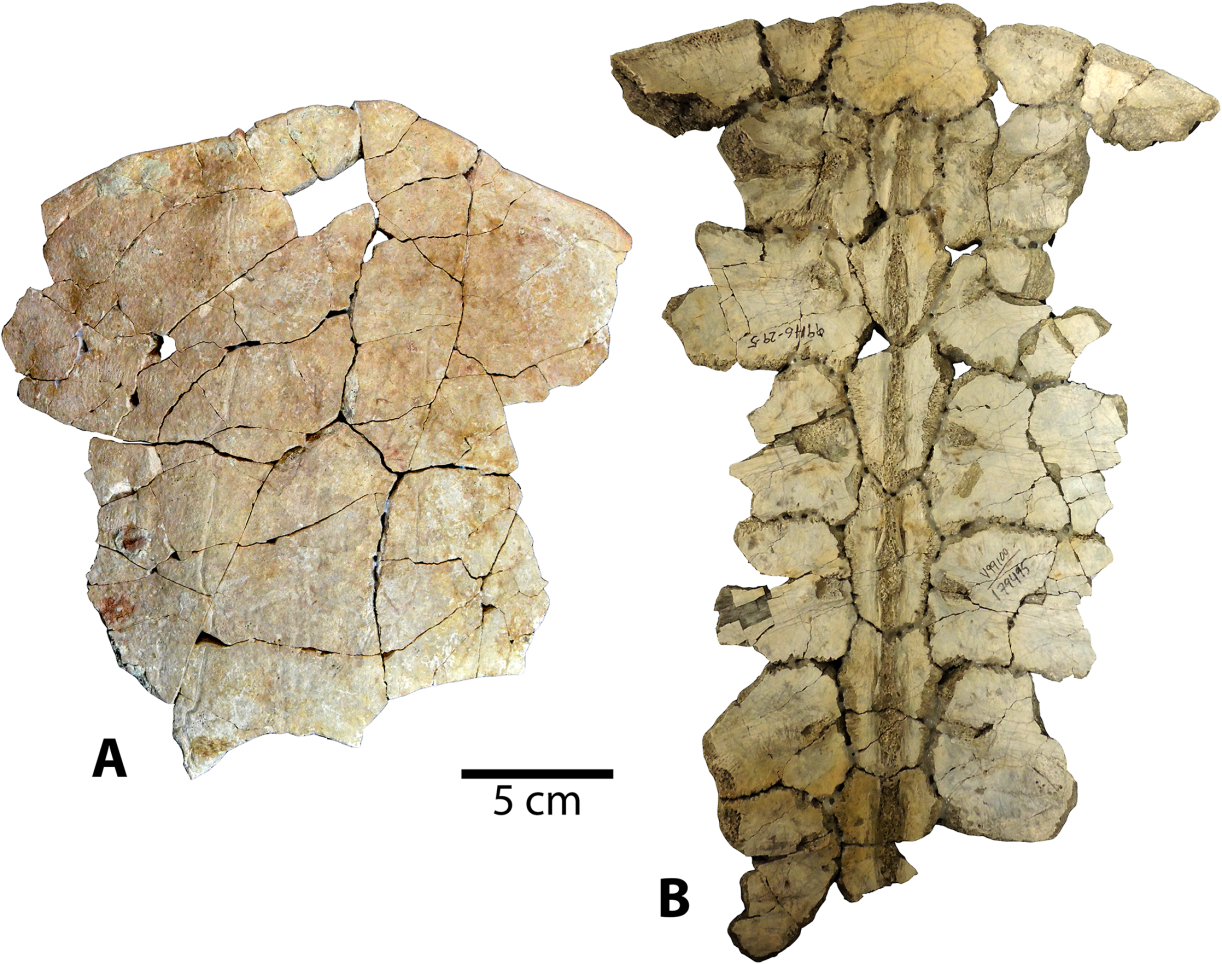
Two Uintan *Baena arenosa* carapace specimens. (A) Dorsal view of UMNH VP 27541 carapace. (B) Ventral view of UCMP 179495 carapace.

**Fig 8:**
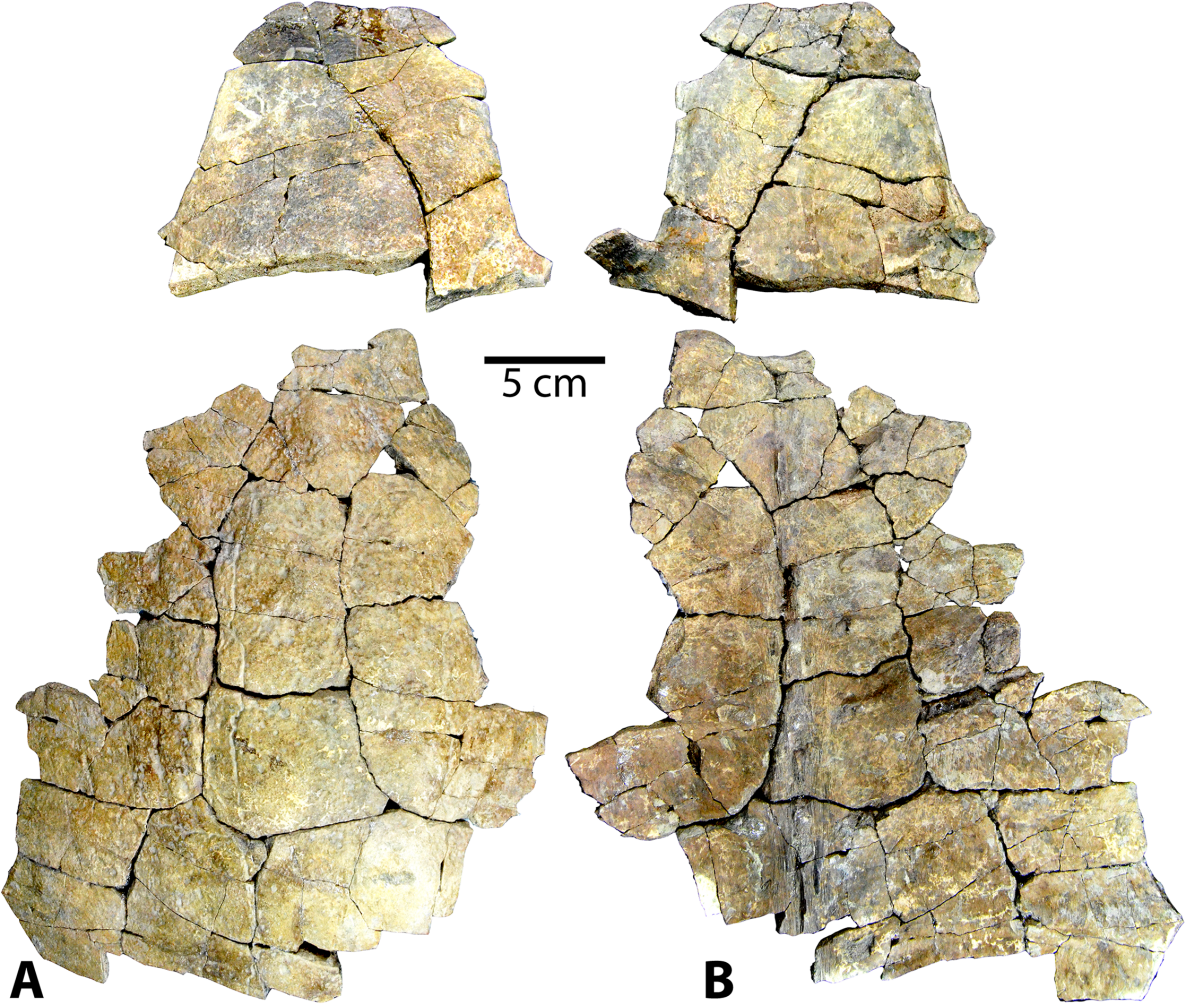
Posterior carapace and plastron sections from Uintan *Baena arenosa* specimen UMNH VP 27192. (A) External view of plastron and carapace. (B) Internal view of plastron and carapace.

**Fig 9:**
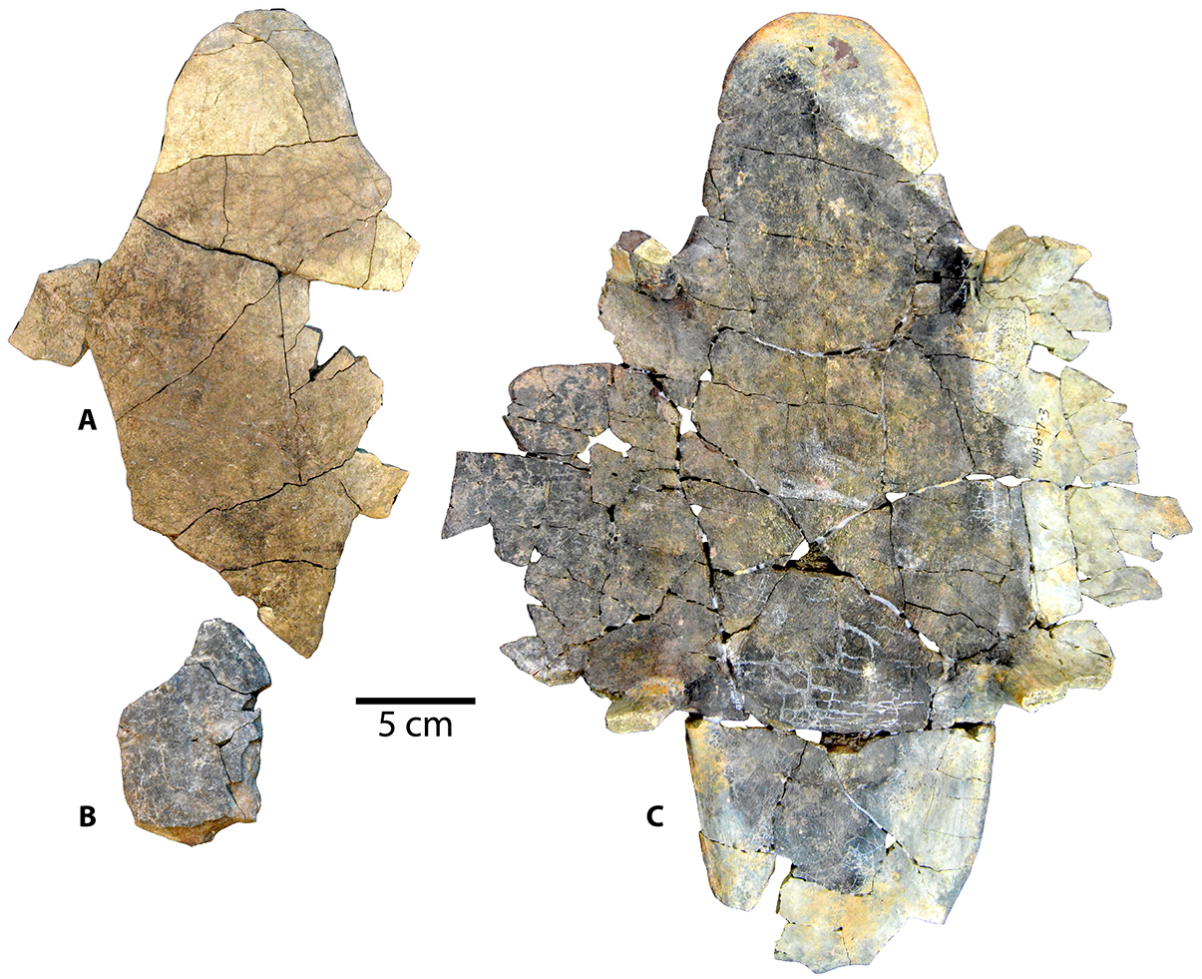
Two *Baena arenosa* plastral specimens from the Uinta Basin, UMNH VP 27085 and UMNH VP 27542. (A) Ventral view of the plastron of UMNH VP 27085. (B) Dorsal surface of midline carapace fragment from UMNH VP 27085. (C) Dorsal view of UMNH VP 27542 plastron.

**Fig 10:**
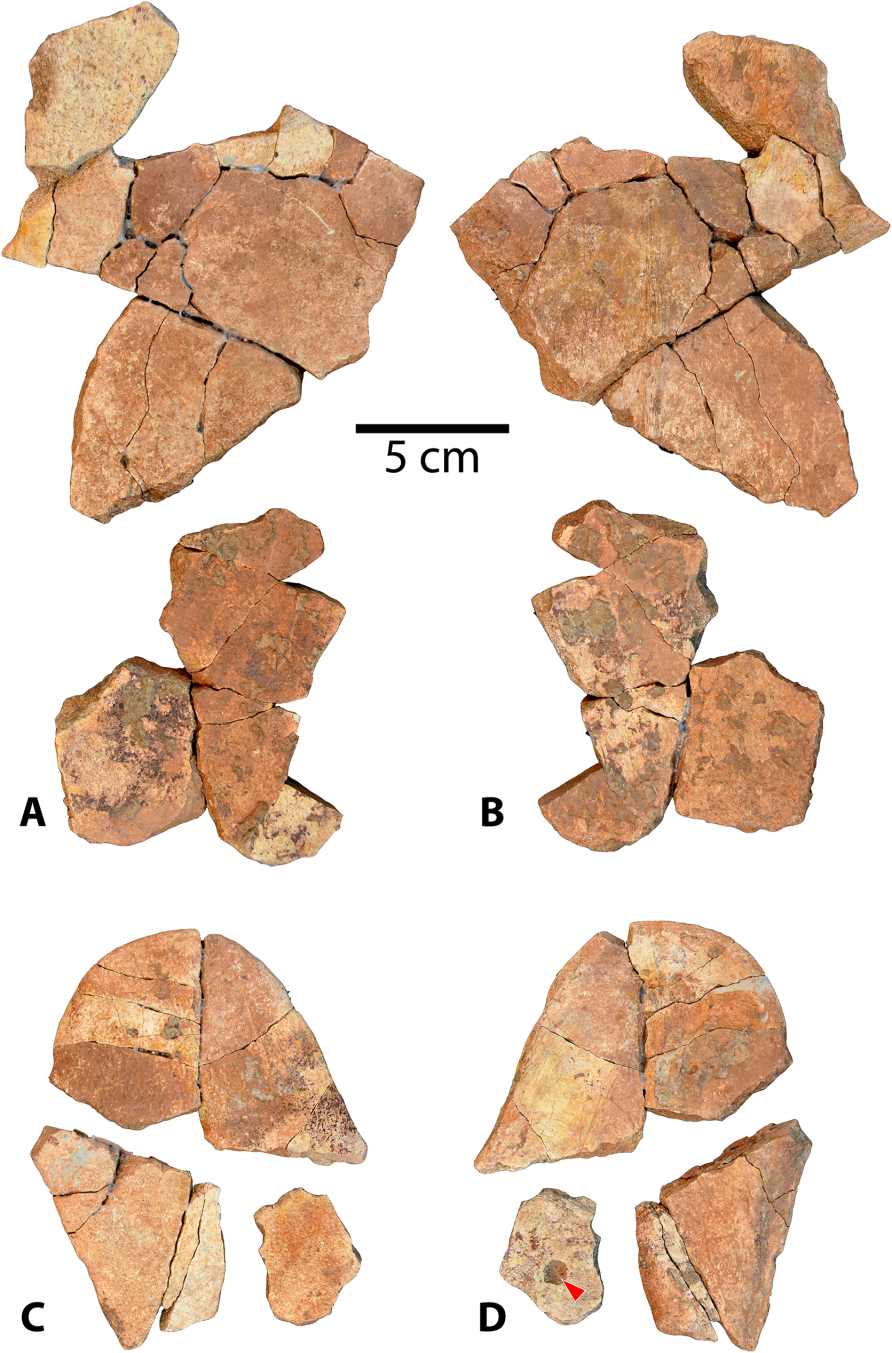
Carapace and plastron fragments of Uintan *Baena arenosa* specimen UMNH VP 27546. (A) Dorsal view of carapace fragments. (B) Ventral view of carapace fragments. (C) Ventral view of plastron. (D) Dorsal view of plastron. Red arrow indicates puncture pit consistent with carnivore tooth mark. Note that this specimen was recovered from the Uinta Formation/Duchesne River Formation contact, making it the youngest baenid specimen described in the present study.

**Fig 11:**
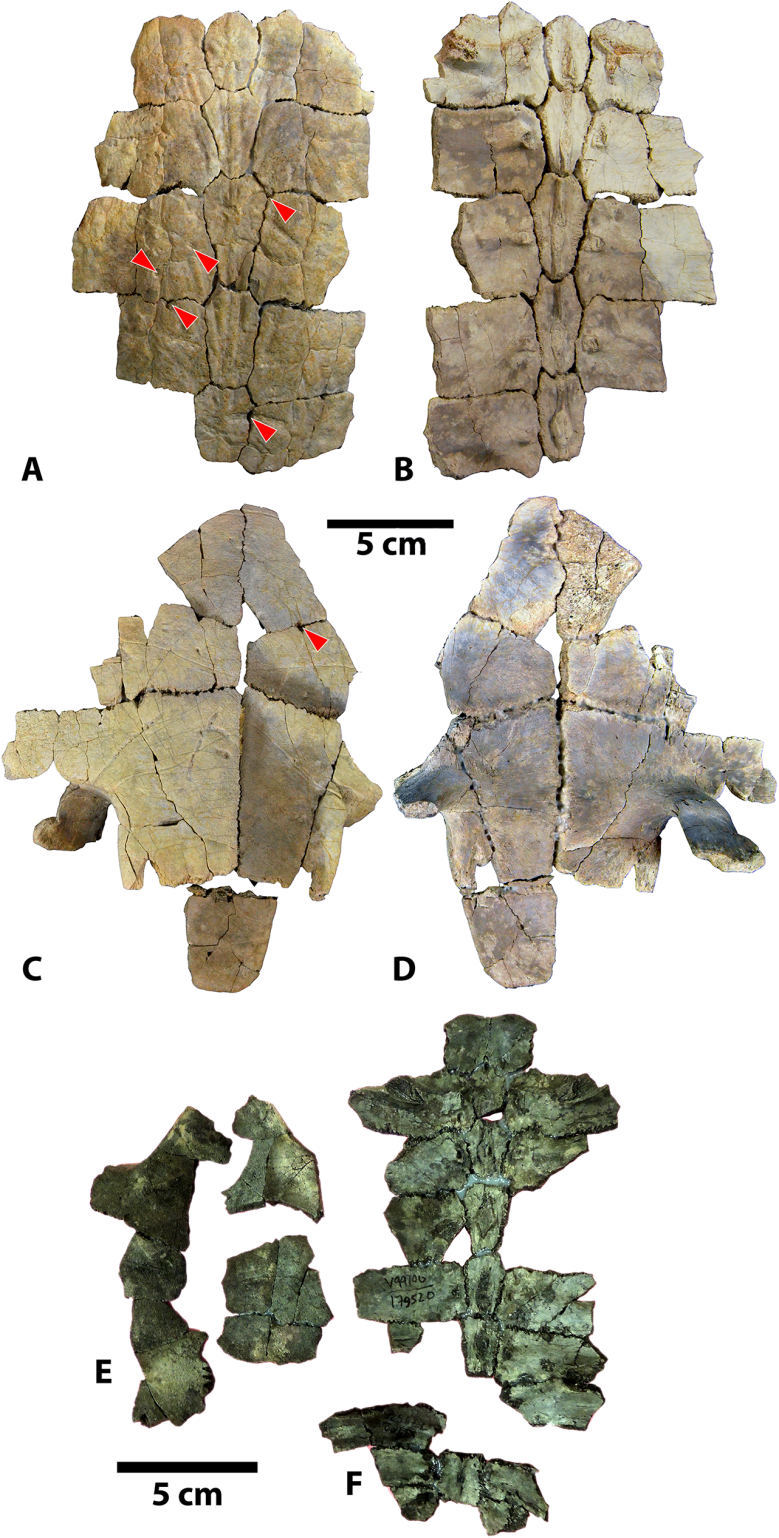
Carapace and plastron fragments of Uintan subadult *Baena arenosa* specimens UMNH VP 27540 and UCMP 179520. (A) Dorsal view of carapace of UMNH VP 27540. (B) Ventral view of carapace of UMNH VP 27540. (C) Ventral view of plastron of UMNH VP 27540. (D) Dorsal view of plastron of UMNH VP 27540. (E) Ventral view of UCMP 179520 plastral fragments. (F) Ventral view of UCMP 179520 carapace. Red arrows indicate puncture pits consistent with carnivore bite marks.

**Fig 12:**
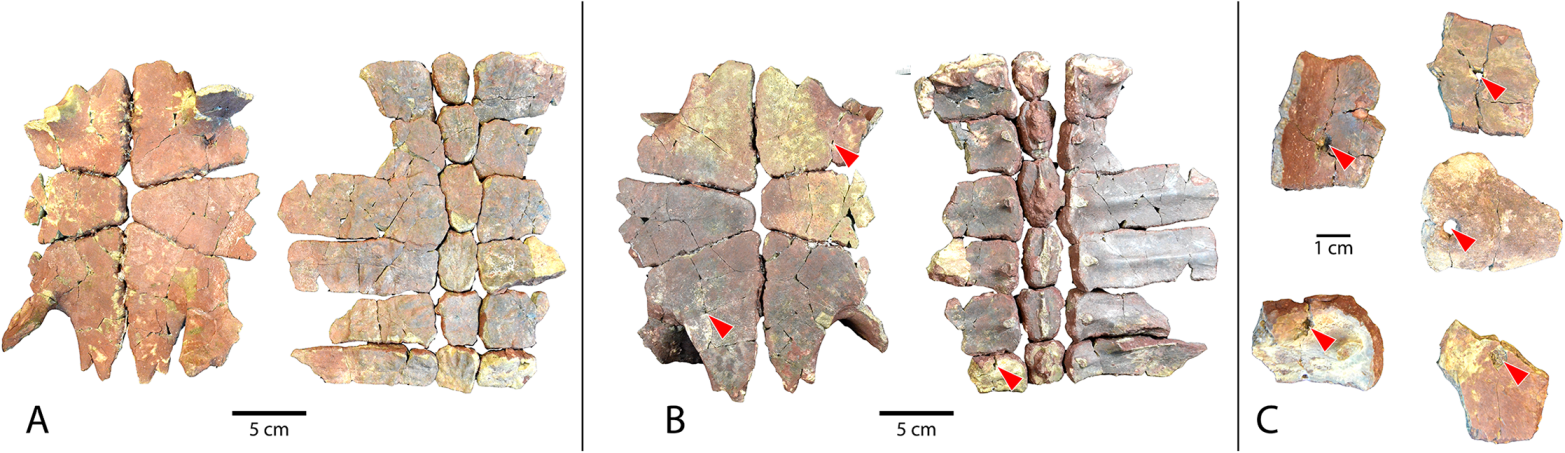
Carapace and plastron fragments of Uintan subadult *Baena arenosa* specimen UMNH VP 27537. (A) Dorsal view of carapace (left) and plastron (right). (B) Ventral view of carapace (left) and plastron (right). (C) Isolated bridge and peripheral elements with evidence of predation. Red arrows indicate puncture pits consistent with carnivore tooth marks.

The new *Baena arenosa* shell material consists of two reasonably complete shells, UMNH VP 27604 and UMNH VP 27191 (Figs 4 and 5), as well as several more fragmentary adult and subadult specimens (Figs 6–12). Specimen UMNH VP 27604 is the most complete baenid shell specimen recovered by our team from the Uinta Formation thus far (Fig 4A and 4B). Only the anterior and posterior tips of the carapace, as well as the left lateral margin, are missing, and the plastron is also essentially complete. Specimen UMNH VP 27191 is a large adult *B*. *arenosa* specimen, including much of the plastron and carapace (Fig 5A–5D). The fusion between bony elements is extensive, such that very few sutures remain visible. The bone of both the plastron and carapace is flatter and thinner than many of the other specimens described here. The dorsal surface of the plastron also contains fewer rugosities and visible muscle attachment markings (Fig 5D). Specimen UMNH VP 27653 is an anterior left portion of a carapace (Fig 6A and 6B). While it is incomplete, the sulci are prominent, and it retains several taxonomically relevant characters. UMNH VP 27539 is a large section of an adult anterior carapace, a fused portion of the anterior plastron, and numerous associated peripheral and bridge fragments (Fig 6C and 6D). Specimen UMNH VP 27543 comprises an anterior plastral lobe, with complete left and right epiplastra, partial entoplastron, and the anterior parts of the hyoplastra, as well as several accompanying nondescript shell fragments. UMNH VP 27545 consists primarily of an extremely weathered plastron, as well as a few carapace fragments. Portions of the left and right hyoplastra, mesoplastra, hypoplastra, and xiphiplastra are present. The anterior margin of the plastron is missing, such that only the posterior parts of the entoplastron and epiplastra are present. Two consecutive partial neurals of unknown position and the medial portions of the adjacent left costals are present. UMNH VP 27541 is a partial weathered carapace and numerous isolated plastral and carapace fragments (Fig 7). It includes fused neurals I-IV, medial portions of costals I-IV, and the anterior margin of the carapace including the nuchal, and first few peripherals on either side. Its dorsal surface is extremely worn such that many of the sulci are not visible. Specimen UMNH VP 27192 is a posterior plastral lobe and posterior carapace (Fig 8A and 8B). Specimen UMNH VP 27542 is a reasonably complete plastron, and a small number of associated float fragments, including several peripherals and bridge elements (Fig 8C). Specimen UMNH VP 27085 consists of a small plastron, missing the posterior region and some of the lateral margins, and a small section of fused neurals (Fig 9A and 9B). The carapace includes a united midline section with fused neurals IV-VII and the associated medial portions of costals IV-VIII, and the pygal/suprapygal region. UMNH VP 27538 includes an abraded, but mostly complete plastron. The lateral aspects of the hyoplastron and hypoplastron and posterolateral portion of the right xiphiplastron are missing, but the remainder of the plastron is present. Specimen UMNH VP 27338 is a weathered partial plastron, missing the posterior and lateral edges. The shell remnants of specimen UMNH VP 27535 (associated with the cranium described above) are unfortunately fragmentary and incomplete (Fig 3G and 3H). On the right side, most of the epiplastron, part of the entoplastron, and the anterior portion of the hyoplastron are present. Only the anteromedial portion of the left epiplastron is present. A fused neural row consisting of three neural elements is present, in addition to two more isolated neural fragments. A partial costal, likely the medial part of costal VIII, is present. It preserves a section of the medial margin of the 4^th^ pleural scale, which appears semicircular. Specimen UMNH VP 27546 includes an anterior plastral lobe, a portion of hypoplastron, and a small midline carapace fragment (Fig 10A–10D).

Shell remains from five new subadult *Baena arenosa* specimens were also discovered. All specimens are diagnosed as subadults due to the presence of sutures and lack of fusion among the individual carapace and plastral elements, as well as their small size. Specimen UCMP 179495 is a larger subadult, consisting of partially fused bones of the midline carapace, including the nuchal, peripherals I-II on either side, neurals I-VI, and the medial portions of the adjacent costals (Fig 7B). Specimen UMNH VP 27540 comprises most of the posterior 2/3 of the plastron, and a large midline portion of the carapace, including a neural row of complete neurals I-V, and the proximal portions of the adjacent costals I-V on both sides, plus part of the right costal VI (Fig 11A–11D). UCMP 179520 includes a section of midline carapace, including the nuchal, neurals I-V and VII, and the medial sections of several contiguous costals, along with some plastral fragments including incomplete portions of both hyoplastra, mesoplastra, and hypoplastra (Fig 11E and 11F). Specimen UMNH VP 27537 includes a partial plastron, the medial portion of the carapace, including neurals I-VI and the medial parts of costals I-VI on both sides, and several isolated bridge and peripheral elements (Fig 12A–12C). Specimen UMNH VP 27547 consists of a small section of right carapace, with three adjacent partial neurals and the proximal portions of the associated costals. UCMP 179496 is a small juvenile consisting of an anterior midline carapace section, including the nuchal, neurals I-III, and costals I, III, and IV on both sides. A large plastral section is also present, including both mesoplastra, left hyoplastron, right hypoplastron and partial right xiphiplastron.

UMNH VP 27604 is an adult *Baena arenosa* specimen, as indicated by complete fusion of the carapacial sutures and size (Fig 4). The midline length of the carapace is approximately 380 mm, which falls within the range of previously described Uintan *B*. *arenosa* specimens (320–390 mm) and below adult *C*. *undatum* specimens (490–520 mm) [26]. UMNH VP 27191 measures 330 mm in midline plastron length, and an estimated midline carapace length of 370 mm (the anterior edge is slightly damaged) (Fig 5). In four specimens, sufficient carapace is preserved to assess the degree of doming. Three (UMNH VP 27191, UMNH VP 27192, UCMP 179283) are moderately domed (Figs 4A and 7A), as in other baenids [6]. However, in the well-preserved UMNH VP 27604, the doming of the carapace is much more pronounced (Fig 4A). The bone of the carapace is generally moderate in its thickness, and noticeably thinner than the plastral bone. However, in one specimen, UMNH VP 27546, the carapace is demonstrably thicker, up to 27 mm (Fig 10). In adult specimens, the carapace lacks a defined mid-dorsal ridge (e.g., Figs 4B, 5A, 6 and 7A). However, in the smaller *Baena* subadults, UMNH VP 27540, UCMP 179520, and UMNH VP 27547, a triple ridge is present, with one midline crest and two parasagittal lines (Fig 11). This condition is present but less pronounced in the larger *Baena* subadults, UMNH VP 27537 and UCMP 179495 (Figs 7B and 11A). A single midsagittal suprapygal element is present, and it divides the pygal in two (Fig 8), as has been described in *Baena arenosa* [55]. The divided pygal and suprapygal surround a pronounced midline caudal notch (Fig 8).

A single large nuchal precedes the first neural, with no evidence of a preneural (Figs 5, 6 and 11). The axillary and inguinal buttresses both reach the costals (Figs 5B and 9), as has been described in other baenids [6]. In the subadult carapaces (UMNH VP 27537, UMNH VP 27540, UMNH VP 27547), the shapes of the unfused neural bones are discernable (Figs 7B, 11 and 12). The neurals vary in shape from oval (neural I) to elongated (neurals II-IV) to roughly rectangular (neurals V-VI). The neurals are generally elongated and wider anteriorly than they are posteriorly (e.g., coffin-shaped), with two sharp parasagittal anterior projections in neurals II-V (Figs 7B and 11 and 12). This morphology creates an intimate articulation between consecutive neurals and with the contiguous costals. Neural I is rounded anteriorly and lacks any anterior parasagittal projections, giving it a more oval shape than neurals II-V. The medial ends of the costals are slightly hooked caudally, creating a tight, wedge-shaped articulation among adjacent costals.

Several Uintan *B*. *arenosa* specimens possess multiple subdivided cervical scales, including UMNH VP 27191, UFH 11739, UCMP 179520, and UCMP 179283 (Figs 5 and 11), a trait not attributed to this species by Joyce and Lyson [6]. However, UMNH VP 27539 displays the expected pattern of a single cervical scale (Fig 7C), suggesting that *B*. *arenosa* is polymorphic for this trait. There is no evidence of a prepleural scale, like that of *C*. *undatum* (Figs 4, 5 and 12; Joyce & Lyson, 2015). There is no evidence of a prepleural scale, the absence of which differentiates *Baena* from other eubaenines [6].

Vertebral I is hexagonal, with a posterior margin that is wider than its anterior margin. Vertebral scales II and III are markedly longer than they are wide, as in other Eubaenines [2, 6], while vertebral IV is only marginally longer than it is wide (Figs 4, 5 and 11). The anterior margin of vertebral V is omega-shaped in the midline, creating an anterior indentation in the posterior margin of vertebral IV (Figs 4 and 8). The fifth vertebral scale extends posteriorly to contribute to the margin of the carapace, as in other baenodds [4, 6]. In the subadult UMNH VP 27537, the anterior sulci of vertebrals II-IV all project to a point at the midline, creating a triangular anterior margin (Fig 12A). The pleural scales are also rather square-shaped. A discontinuous section of right peripherals and the lateral portion of a posterior bridge element are present in one adult specimen (UMNH VP 27192), demonstrating a square-shaped marginal scale (Fig 8). In UMNH VP 27604, the pleural-marginal sulci are deep and pronounced, and the marginals are tall and almost vertically oriented due to the height of the dome-shaped carapace (Fig 4A).

In UMNH VP 27191, vertebrals II-IV are rectangular (longer than wide) (Fig 5A and 5A'), as in other Eubaenines [2, 6]. Vertebral V is incomplete posteriorly, so it is not possible to determine whether it contributes to the posterior margin of the shell. The cervical scale is subdivided into several small cervicals (Fig 5A), as illustrated by Gaffney [2]. Prepleural scales, which are typically present in *C*. *undatum* and absent in *B*. *arenosa* [6], are absent in UMNH VP 27191 (Fig 5A).

The shells of subadult *B*. *arenosa* specimens UMNH VP 27537 and UMNH VP 27540 demonstrate evidence of apparent predation (Figs 11 and 12). Several deep, round puncture pits are visible on the bridge, plastron, and carapace of UMNH VP 27537. The most prominent pits are those on the bridge elements, which pass completely through the bone in some cases (Fig 12). The ventral surface of the plastron and dorsal carapace of UMNH VP 27540 are also perforated in several locations by conspicuous puncture pits (Fig 11).

Attached to the caudo-ventral surface of the carapace in UNMH VP 27191, are the articulated centra of four caudally positioned vertebrae: Dorsal vertebrae 8–10 (D8-10) and Sacral vertebra 1 (S1) (Fig 5B and 5B'). The vertebral centra of the final three dorsal (thoracic) vertebrae are broad and cylindrical (Fig 5B'). The proximal portions of the articulated rib heads are present along the left side of the vertebral series. The centrum of S1 is wider than the centra of the dorsal vertebrae, and the lateral extension is cylindrical and projects caudolaterally from the body on the right side (Fig 5B').

**Fig 13:**
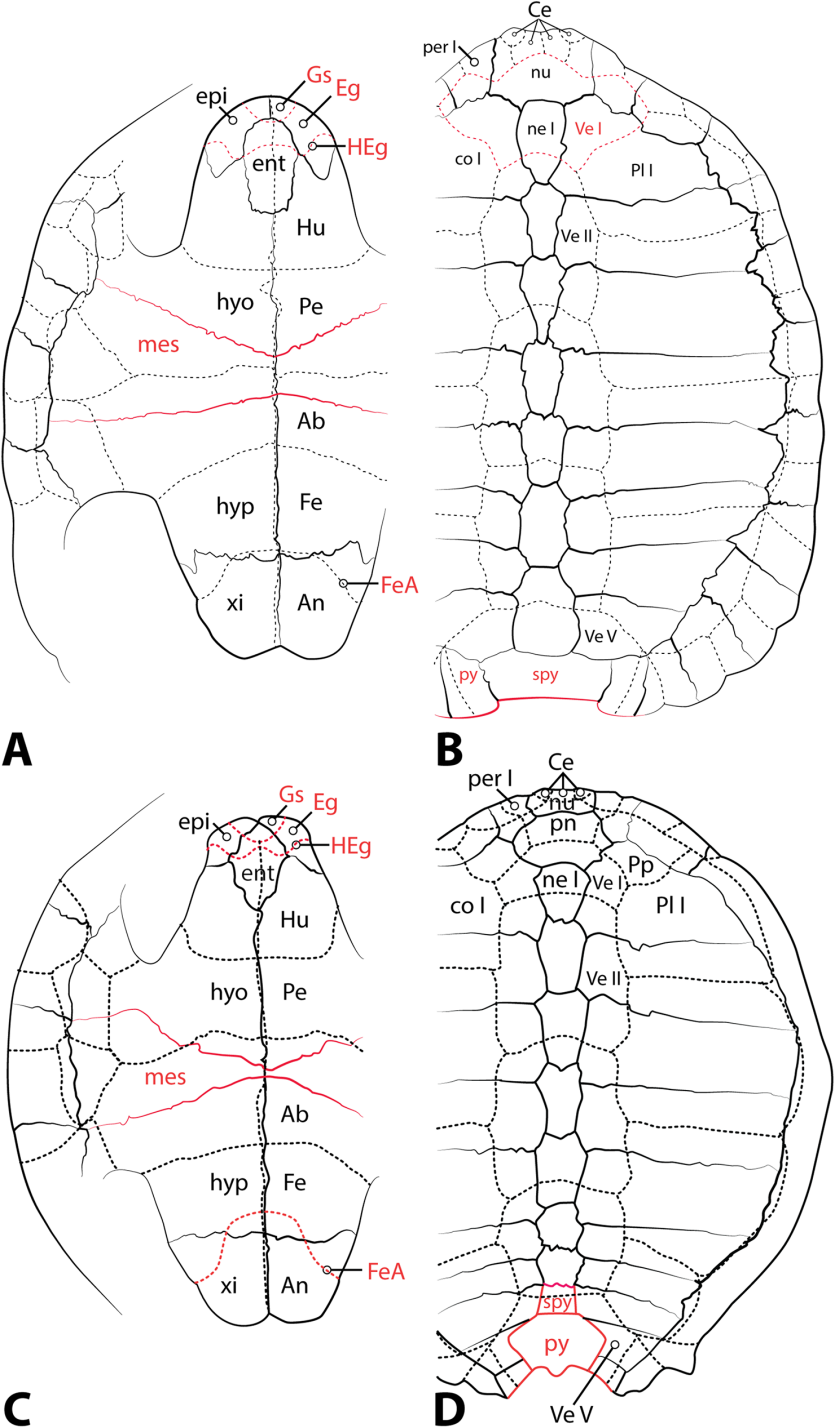
**Schematic representations of morphological traits in newly described Uintan *Baena arenosa* (A-B) and *Chisternon undatum* (C-D) specimens.** (A) Ventral plastron of Uintan *B*. *arenosa*. (B) Dorsal carapace of Uintan *B*. *arenosa*. (C) Ventral plastron of Uintan *C*. *undatum*. (D) Dorsal carapace of Uintan *C*. *undatum*. Differences from Gaffney (1979) [46] indicated in red. Uintan baenid specimens generally differ from previously described *B*. *arenosa* and *C*. *undatum* morphology in having: sigmoidal humeral-extragular sulci, multiple cervical scales, and small subtriangular gular scales. Abbreviations: Ab = Abdominal scale; An = Anal scale; Ce = Cervical scale; co = costal; Eg = Extragular scale; epi = epiplastron; ent = entoplastron; Fe = Femoral scale; FeA = Femoral-Anal sulcus; Gs = Gular scale; HEg = Humeral-Extragular sulcus; Hu = Humeral scale; hyo = hyoplastron; hyp = hypoplastron; mes = mesoplastron; ne = neural; nu = nuchal; Pe = Pectoral scale; per = peripheral; Pl = Pleural scale; pn = preneural; Pp = Prepleural scale; py = pygal; spy = suprapygal; Ve = Vertebral scale; xi = xiphiplastron. Following Gaffney (1979) [46], abbreviations for scales are capitalized, while those for bony elements are indicated with lowercase.

The new plastral specimens of Uintan *Baena arenosa* expand the range of known variation within the species, while still retaining the primary characteristics expected within the Baenidae (Figs 5–13). For the measurable adult *B*. *arenosa* specimens, the maximum plastron length ranges from 330–380 mm in length, placing them securely within the published range for *Baena arenosa* (under 400 mm) [26], and below that of known adult *C*. *undatum* specimens (420–480 mm) [26]. Even in some of the smaller adult specimens (e.g., UMNH VP 27085), the plastral elements are typically completely fused, providing further support that these specimens are attributable to *B*. *arenosa*, because the sympatric *C*. *undatum* often retains sutures into adulthood [26].

The sulci are prominent, and are visible exclusively on the ventral surface of the plastron, indicating that the plastral scales did not overlap to the dorsal surface, as in other Baenids [6, 55]. The anterior plastral lobes are relatively narrow, but generally rounded, in contrast to the truncated, subtriangular shape typically observed in *C*. *undatum* [6, 55]. In one specimen (UMNH VP 27538), the epiplastra are indented anterolaterally, causing the anterior margin of the plastron to appear knob-shaped. There is no evidence of epiplastral processes. In the subadults UMNH VP 27537, UCMP 179520, UCMP 179496, and UMNH VP 27540, patent sutures demonstrate the shapes of the individual plastral bones (Figs 11 and 12). In all subadults, the mesoplastra contact each other broadly at the midline, as is characteristic of *B*. *arenosa* [6, 56].

The gular scales are small, and for the most part, roughly ovoid. In some specimens (e.g., UMNH VP 27543), together the left and right gular scales form a semicircle. The extragular scales are substantially larger than the gular scales, and contact each other broadly in the midline posterior to the gular scales (Fig 5C), as is typical of the Baenodda clade [4, 6]. The humeral-extragular sulcus is distinctly sigmoidal in many specimens (i.e., UMNH VP 27539, UMNH VP 27541, UMNH VP 27543, UCMP 179283; Figs 4 and 6), a trait which has been described in other eubaenines but not typically thought to characterize the Uintan baenids, *Baena* or *Chisternon* [6]. In some specimens (i.e., UMNH VP 27338, UMNH VP 27535, UMNH VP 27538, UMNH VP 27542; Fig 13), the sigmoidal shape is definitively present, but more gradually sloping. Only one specimen displays a non-sigmoidal shape: UMNH VP 27085 has a straighter, subtle inverted omega shaped humeral-extragular sulcus thought to characterize *B*. *arenosa* (Fig 9A) [6].

The posterior plastron is generally broad in comparison to the narrow anterior lobe. The hypoplastra are dorsoventrally thickened at the midline. The posterior buttresses are robust, although they are flattened in the juvenile UMNH VP 27537 (Fig 12). The posterior margins of the xiphiplastra curve slightly anteriorly at the midline, creating a very subtle caudal notch (UMNH VP 27192, UMNH VP 27191, UMNH VP 27542, UCMP 179283; Figs 4 and 7A). In most specimens, the femoral-anal sulcus is omega-shaped, and crosses the xiphiplastron-hypoplastron suture (Fig 5C), as in other baenodds [4, 6]. In one specimen (UMNH VP 27191), the femoral-anal sulcus appears quite straight compared to other Uintan baenids, and does not appear to extend onto the hypoplastron (Fig 5C); however, the sutures are obliterated in this specimen, so the hypoplastral-xiphiplastral boundary can only be estimated.

*CHISTERNON UNDATUM* Leidy, 1873 [57]

**Fig 14:**
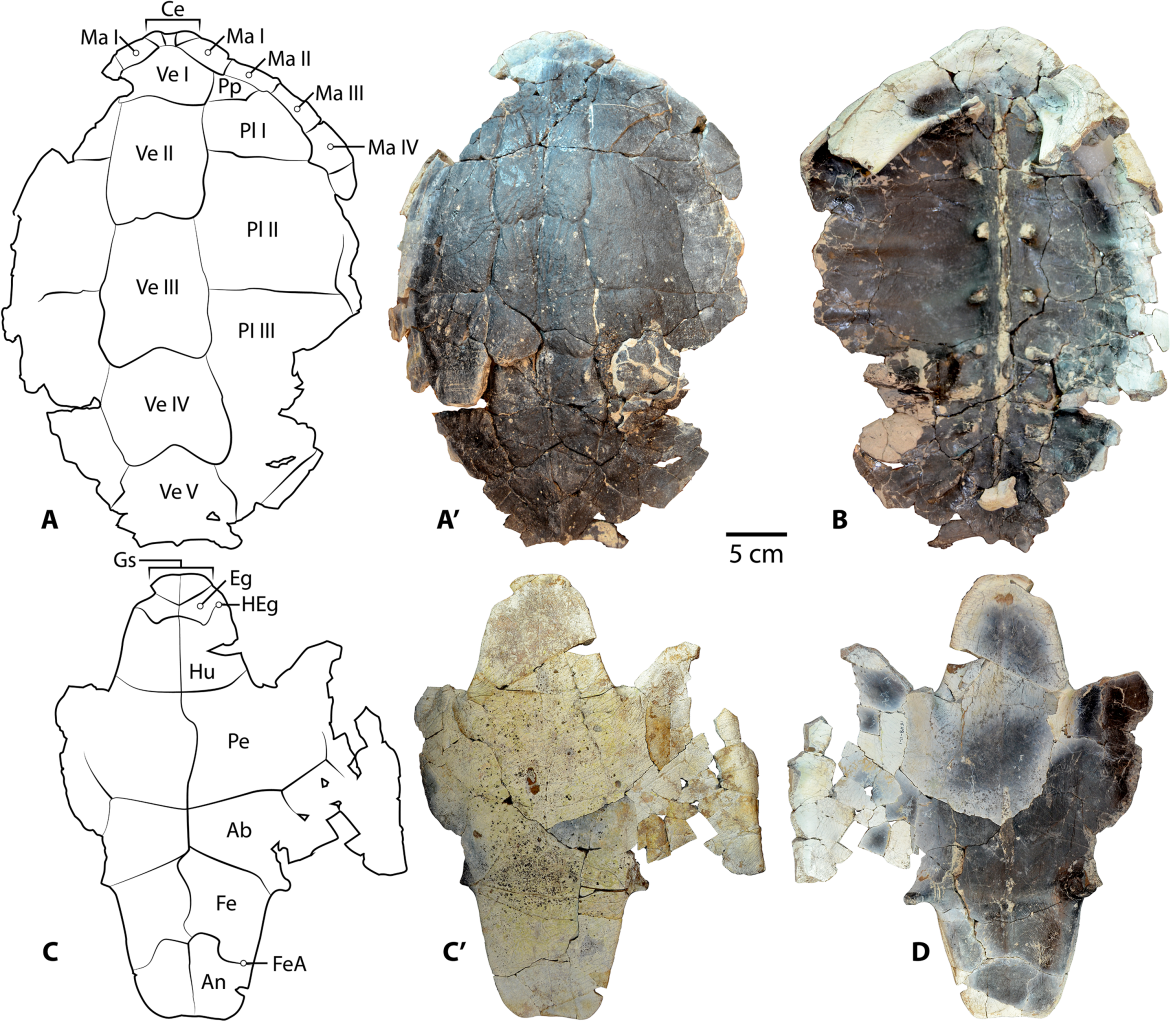
Carapace and plastron of Uintan *Chisternon undatum* specimen UMNH VP 27554. (A) Dorsal view of carapace. (B) Ventral view of carapace. (C) Ventral view of plastron. (D) Dorsal view of plastron. Abbreviations: Ab = abdominal scale; An = anal scale; Ce = cervical scale; Eg = extragular scale; Fe = femoral scale; FeA = femoral-anal sulcus; Gs = gular scale; Hu = humeral scale; HEg = humeral-extragular sulcus; Ma = marginal scale; Pe = pectoral scale; Pl = pleural scale; Ve = vertebral scale.

**Fig 15:**
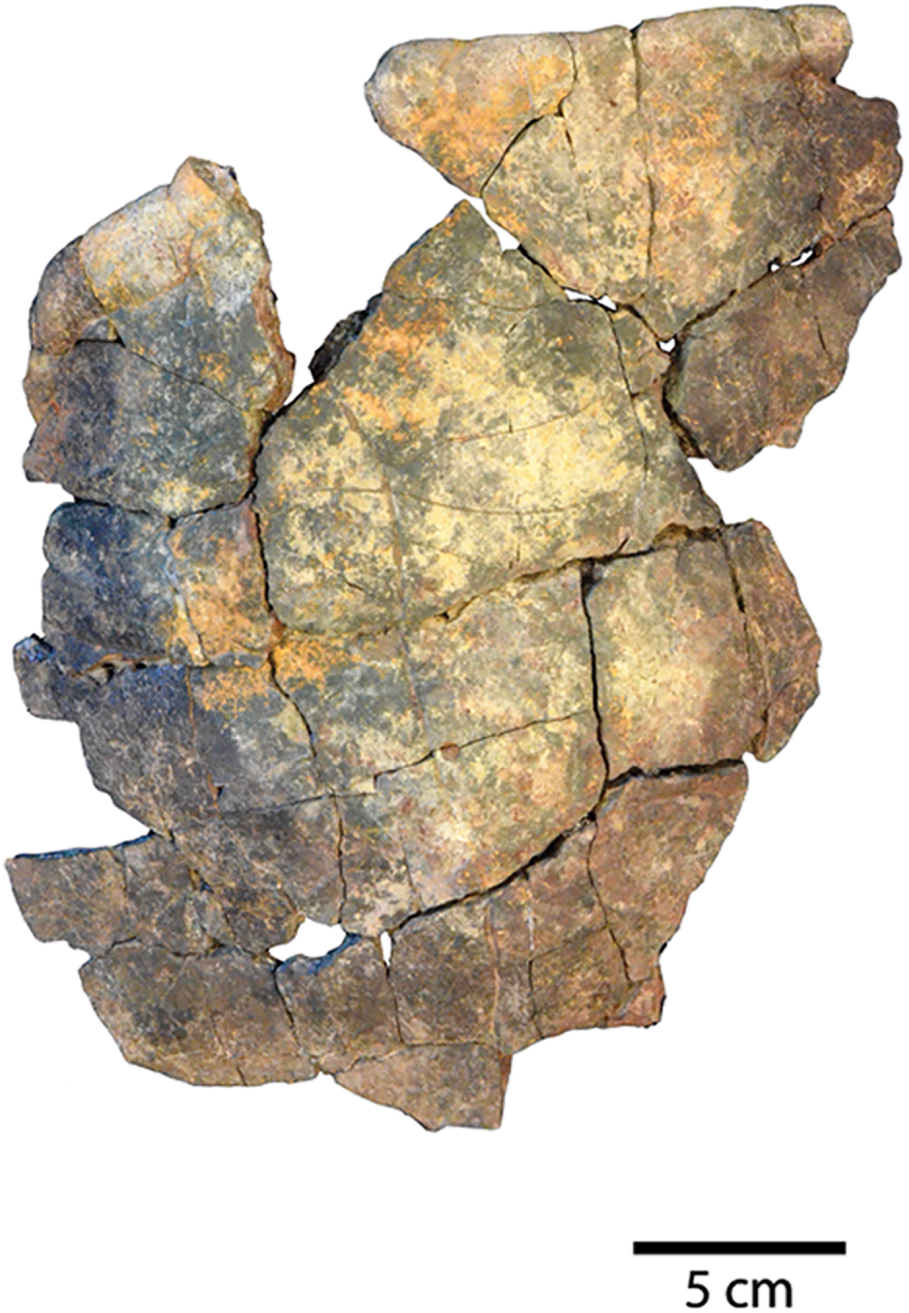
Partial Uintan *Chisternon undatum* specimen, UCMP VP 27652. Dorsal view of carapace.

**Fig 16:**
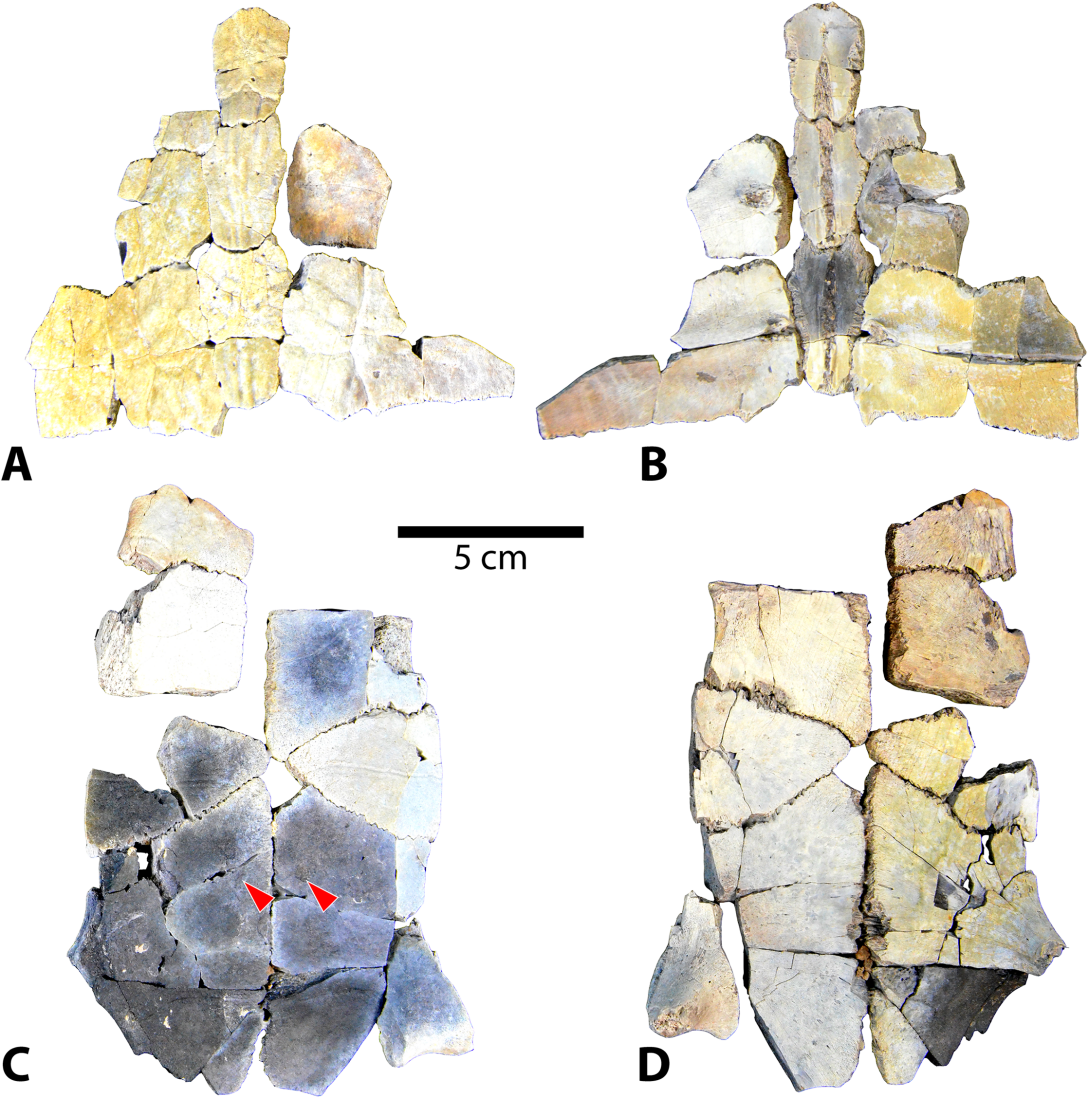
Carapace and plastron fragments of Uintan subadult *Chisternon undatum* specimen UMNH VP 27544. (A) Dorsal view of carapace. (B) Ventral view of carapace. (C) Ventral view of plastron. (D) Dorsal view of plastron. Red arrows indicate puncture pits consistent with carnivore tooth marks.

Referred specimens: The new *Chisternon undatum* material (Figs 14–16) includes one very complete shell (UMNH VP 27554), and four incomplete specimens, including one juvenile (UMNH VP 27544). Specimen UMNH VP 27554 consists of a sizeable, almost complete plastron and carapace in excellent condition, such that many regions and features not preserved in other Uintan specimens are observable (Fig 14A–14D). The plastron of this specimen is in exceptional condition, with only portions of the right lateral margin missing, plus a few additional minor chips in the epiplastra and xiphiplastron (Fig 14C and 14D). The carapace consists of a greater number of small fragments than the plastron, but it is still mostly reconstructable and the majority of it is present. Specimen UMNH VP 27652 is the partial left anterior carapace and associated hypoplastral midline fragment, preserving the base of the left posterior buttress (Fig 15). It is quite weathered, and little diagnostic morphology is preserved. UMNH VP 27319 is a section of anterior midline carapace. UMNH VP 26729 is a large, fragmentary specimen consisting of two fused sections of midline carapace and two sections of plastron. The external surface is badly weathered, rendering the sulci virtually imperceptible. The carapacial bone of UMNH VP 26729 is extremely thick. While this specimen is quite fragmentary, it is diagnosed as *C*. *undatum* based on the large size and extensive thickness [26]. Specimen UMNH VP 27544 is a juvenile *Chisternon undatum*, which includes a partial neural row with a few adjacent costals, a partial plastron, and numerous undiagnostic carapace and plastron fragments (Fig 16). The plastron of UMNH VP 27544 is not as well-preserved as the carapace, but much of it is present, including most of the hyoplastra, mesoplastra, and right hypoplastron. Sutures are patent in some areas of both the plastron and carapace in several of the presumed adult *C*. *undatum* specimens described here (UMNH VP 27554, UMNH VP 27652, UMNH VP 26729; Figs 14 and 15), despite their large body size. Hutchison [26] notes the retention of some visible sutures longer into adulthood as a differentiating feature of *C*. *undatum* compared to *B*. *arenosa*.

### Description and comparison

#### Carapace

The most complete carapace, UMNH VP 27554, is a large specimen, with a midline carapace length of 440 mm (Fig 14A and 14B). This magnitude places the specimen securely within the body size of *Chisternon undatum*, the larger of the two baenid species from the Uinta Formation [26]. Additionally, many of the carapacial and plastral characters associated with *C*. *undatum* are also present in this specimen. Intriguingly though, its maximum plastral length is relatively shorter at 367 mm, which falls at the threshold between the ranges for *B*. *arenosa* and *C*. *undatum* [26]. The original curvature of the carapace is maintained, and the shell profile is elongated and substantially flatter than the similarly preserved *B*. *arenosa* specimen, UMNH VP 27604 (Figs 3A and 14A'). The other incomplete carapace representatives (UMNH VP 27319, UMNH VP 27652, UMNH VP 27544) also appear relatively flattened anteriorly (Figs 15 and 16), suggesting a low dome, although it is not possible to directly measure the curvature in these partial specimens. The nuchal area in UMNH VP 27554 (Fig 14B) and UMNH VP 27319 is fused, making it difficult to definitively assess the presence of a preneural bone; however, the distance between the anterior tip of the shell and the first neural is quite long (50.1 mm and 48.9 mm, respectively), substantially longer than in UMNH VP 27191 (32.5 mm), a *B*. *arenosa* specimen of similar body size (Fig 5). This increased distance suggests the presence of multiple bones anterior to the first neural in UMNH VP 27554 and UMNH VP 27319.

There is a subtle mid-dorsal ridge that runs the length of the available neural series in UMNH VP 27554 (Fig 14A). However, the incomplete UMNH VP 27652, UMNH VP 27319, and UMNH VP 26729 all lack any apparent midline ridge (Fig 15). In the subadult UMNH VP 27544, this crest expands out into a triple ridge in some places, with two parasagittal lines running parallel to the midline elevation (Fig 16A). This condition mirrors that seen in subadult *B*. *arenosa* specimens UMNH VP 27537, UMNH VP 27540, UCMP 179520, and UMNH VP 27547 (Figs 11 and 12). In UMNH VP 26729, visible sutures of two neurals of unknown position reveal a very rectangular shape of these elements. In the subadult UMNH VP 27544, the neural bones are coffin-shaped (Fig 16A and 16B), and neural I is subrectangular, while neurals II and III are elongated and contain two parasagittal anterior projections. The pygal is divided into two separate elements by the suprapygal (Fig 14A and 14B), as has been described in other Uintan baenids [55]. The posterior margin of the suprapygal is curved and concave anteriorly, creating a caudal carapacial notch, with the exception of a small, posterior midline projection (Fig 14A and 14B).

The cervical scale is divided into multiple small cervical scales (Figs 14A, 14B and 15), as in other Eubaenines other than *Baena* [2]. A prepleural scale, an archetypal *C*. *undatum* trait [6, 26], is present (Figs 14A, 14A' and 15). Vertebral I of UMNH VP 27539 has the shape of a flattened pyramid, constricted anteriorly with its lateral borders flaring more laterally as they course caudally, such that the scale is wider caudally than it is cranially. Vertebrals II-IV are longer than they are wide (Fig 14A and 14A'), as in Eubaenines [2, 6]. Vertebral V contacts the posterior margin of the carapace, interrupting the ring of marginal scales (Fig 14A and 14A'), as in baenodds [4, 6].

#### Plastron

The measurable maximum plastral lengths in these new *C*. *undatum* specimens range from 367–400 mm. The anterior lobe of the plastron is truncated, and triangular-shaped compared to the *B*. *arenosa* specimens described herein (Fig 14C and 14D). The sulci do not extend onto the dorsal surface, as in other baenids [6, 55]. There is a thick midline ridge coursing antero-posteriorly along the dorsal surface of the entoplastron (Fig 14C). The mesoplastra are triangular and converge to contact each other at a small point at the midline (Fig 14). This mesoplastral shape typifies *C*. *undatum* to the exclusion of other baenids [6]. In the subadult UMNH VP 27544, the right and left mesoplastra are separated by a midline fontanelle, although these elements expand in adulthood to articulate with one another (Fig 15C and 15D). The anterior buttresses are prominent, and contact the anterior costals extensively, as in other baenids (Figs 14 and 15) [6]. The cranial opening is consequently quite narrow in the single specimen in which it can be fully assessed (UMNH VP 27554; Fig 14).

The gular scales are moderately-sized and subtriangular (Figs 14C, 14C'–16), as in *Eubaena hatcheri* [8]. However, the extragular scales contact each other broadly at the midline posterior to the gular scales (Fig 14C), as characterizes baenodds [4, 6], albeit not as extensively as in the *B*. *arenosa* specimens. The humeral-extragular sulcus varies in its form among the *C*. *undatum* specimens. In UMNH VP 27554, the humeral-extragular sulcus is sigmoidal (Fig 14C), as in other Uintan specimens (such as *Baena* specimens UMNH VP 27539, UMNH VP 27543, UMNH VP 27541). However, UMNH VP 26729 demonstrates the more classic eubaenine condition of a relatively straight course. The femoral-anal sulcus, however, is omega-shaped and continues onto the hypoplastron (Figs 14 and 16), as in other baenodds [4, 6]. There is a slight, sloping xiphiplastral notch (Fig 14 C and 14C'). The interfemoral sulcus is deep and relatively straight (Fig 14C).

### Phylogenetic analyses

These newly described Uintan specimens of *B*. *arenosa* and *C*. *undatum* add previously undocumented morphological variation into the hypodigms of both species. With the addition of these new specimens, we were able to code 7 characters previously coded as unknown (“?”) [36] for one or both Uintan baenid species (characters 27, 29, 46, 48, 55, 57, 68), and update the coding for 3 additional characters (40, 64, 65). Thus, we performed a phylogenetic analysis to determine whether these changes in character states resulted in a different understanding of baenid phylogenetic relationships.

**Fig 17:**
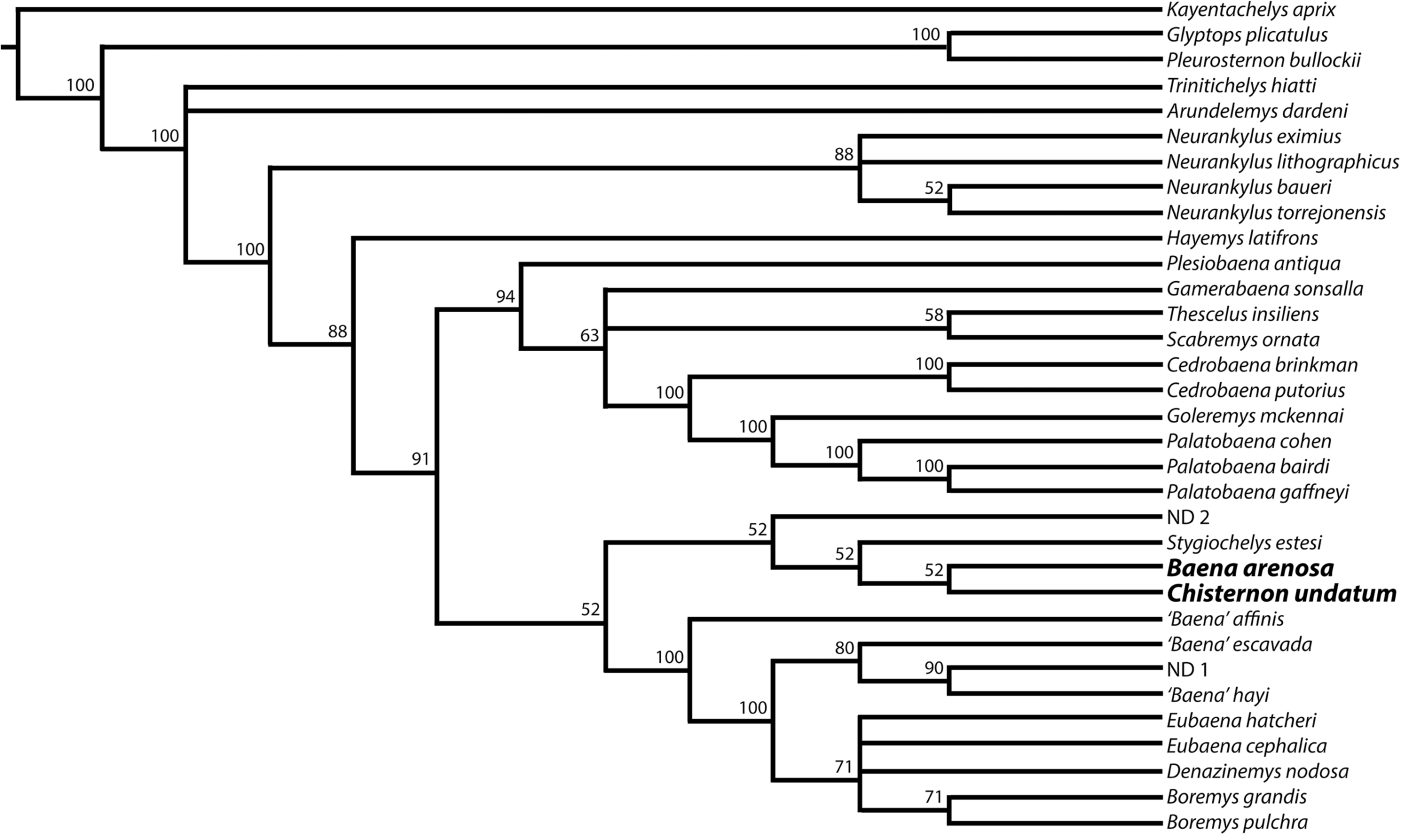
Results of phylogenetic analysis of Baenidae, incorporating newly described variation in Uintan *Baena arenosa* and *Chisternon undatum*. Majority rule consensus cladogram of 72 most parsimonious trees. Percentages of trees supporting each node are indicated. *B*. *arenosa* and *C*. *undatum* were found to be sister taxa in 52% of the minimum length trees.

There were 72 most parsimonious trees recovered from the phylogenetic analysis, with a length of 175 steps (CI = 0.474, RI = 0.526). A majority-rule consensus cladogram of these minimum length trees is illustrated in Fig 17. In a slight majority of trees (52%), *Baena arenosa* and *Chisternon undatum* were positioned as sister taxa, with *Stygiochelys estesi* as their outgroup. However, in most of the remaining 48% of trees, *C*. *undatum* was positioned as the sister taxon to *S*. *estesi*. This clade was supported by five derived character state changes.

## Discussion and conclusions

The recently recovered baenid specimens documented here represent among some of the latest surviving specimens from this clade described to date, including the penultimate documented cranial remains. These late-surviving representatives expand the range of known morphological variation in both taxa, *Baena arenosa* and *Chisternon undatum*, confirm the sister-taxon relationship between them, and revise the list of characters currently used to define these species and other taxonomic categories within the Baenidae. Six new subadult specimens also provide insight into the ontogeny, life history, and predation pressures of Uintan baenids.

### Morphological variation

#### Cranial morphology

The new *Baena arenosa* partial skull UMNH VP 27535 exhibits morphological patterns that are generally consistent with that of other *B*. *arenosa* crania (Fig 2). However, a few notable variations were observed, especially in the basicranial region. In UMNH VP 27535, the tuberculum basioccipitale flare out laterally, and their tips are indented. This morphology contrasts with that of the other Uintan *B*. *arenosa* specimen CM 2956, in which the tuberculum basioccipitale are oriented almost directly caudally, and the tips are smooth and slightly rounded. The tuberculum of UMNH VP 27535 also extend much further caudally (~4.8 mm from the rest of the basicranium) and flare more widely than in the subadult *B*. *arenosa* MCZ 4072, in which they hardly extend beyond the margin of the body of the basioccipital. The condylus occipitalis of UMNH VP 27535 is crescent-shaped and concave dorsally, in contrast to the oval shape of CM 2956.

The two foramina posterius canalis carotici in UMNH VP 27535 are round, large, and contained entirely within the pterygoid. This condition is unlike that of most baenids, in which these foramina lie within the basisphenoid-pterygoid suture. However, while an interesting trait, variation in position and size of this foramen likely has limited taxonomic implications [48]. The large size of this foramen implies a sizeable arteria carotica interna, which provides the primary blood supply to the brain.

While the temporal region of UMNH VP 27535 is incomplete, the preserved portion of the fossa temporalis superior suggests a minor degree of temporal emargination, similar to other *B*. *arenosa* specimens, including CM 2956, USNM 17998, and AMNH 5971 [2]. Gaffney [2] and Joyce and Lyson [6] attribute this character to the genus *Baena*, and consider it a differentiating factor between it and the contemporaneous *Chisternon*. A reduction in temporal emargination evolved independently in several baenid lineages after the K/T Boundary [37]. Gaffney [48] suggested that *B*. *arenosa* nasal bones may be either fused to the frontals or lost entirely. However, in UMNH VP 27535, a suture is visible between the nasal and frontal on the left side, suggesting that these elements are separate bones in this specimen.

#### Shell morphology

These newly discovered shell specimens of *Baena arenosa* and *Chisternon undatum* extend the recognized morphological variation in these species, and call into question the validity of several characters that employed in taxonomic assessments within Baenidae. First, baenid shells are typically described as moderately domed [2, 6]; yet, in the case of *Baena arenosa* specimen UMNH VP 27604, the degree of carapacial doming is quite pronounced (Fig 4A). The steep lateral margins of the carapace created by this doming result in peripherals that are nearly vertically oriented and costals that are extremely curved. This contrasts with the more classically less domed Uintan *B*. *arenosa* specimens UMNH VP 27191 and UMNH VP 27192 (Figs 5–8), and *C*. *undatum* specimen UMNH VP 27554 (Fig 14), although some of this flatness may be the result of post-depositional deformation.

*B*. *arenosa* and *C*. *undatum* have both been described as lacking a sigmoidal humeral-extragular sulcus [6]. However, in the majority of our specimens for which this morphology was preserved (n = 10), we observed either a distinctly sigmoidal or gently sigmoidal sulcus (Figs 3, 5, 10 and 13). Only two specimens deviate from this pattern: one *B*. *arenosa* specimen (UMNH VP 27085) in which the humeral-extragular sulcus is a subtle inverted omega shape (Fig 8), and one *C*. *undatum* (UMNH VP 26729) with the expected straight humeral-extragular sulcus morphology. Additionally, in one *B*. *arenosa* specimen (UMNH VP 27191), the femoral-anal sulcus lacks the distinguishing omega shape of baenodds, and does not appear to extend onto the hypoplastron (Fig 5). While the remainder of the Uintan baenid specimens described herein display the expected omega configuration of this sulcus, this uncharacteristic specimen highlights existence of variation within the species, and moderates the synapomorphic status of this trait within the Baenodda.

In several Uintan *B*. *arenosa* specimens that adequately preserve the anterior carapace (e.g., UMNH VP 27191, UMNH VP 27541, UMNH VP 27653, UFH 11739), the cervical scale is divided into multiple cervicals (Figs 4C, 5A and 6A). This condition is consistent with AMNH 5977 and 5900 as depicted by Gaffney [2], who classified these specimens as *B*. *arenosa*, and with other eubaenine taxa. However, it should be noted that given the recent resurrection of “*B*.*” affinis* [6], the *B*. *arenosa* hypodigm as described by Gaffney should best be viewed as a chimera. This morphology contrasts with Joyce and Lyson [6], who describe *Baena* as being differentiated from other eubaenines by possessing a single, undivided cervical scale. In our sample, only UMNH VP 27539 appears to display the single cervical scale morphology (Fig 6C). The type specimen of *B*. *arenosa*, USNM 103, is unfortunately missing the anterior border of the carapace [56]. Hay [8] also described multiple cervical scales in his “*Baena riparia*” (AMNH 106), specimens which are now subsumed within *B*. *arenosa*. Thus, it appears that many specimens now included in the *B*. *arenosa* hypodigm, including the new Uintan fossils described here, possess a divided cervical scale. This suggests that this character is variable within the species, or misinterpreted by Joyce and Lyson [6]. Since multiple cervical scales are considered a synapomorphy of the Eubaeninae [2], this finding is not surprising. Furthermore, the baenid *Boreyms pulchra* has also been described as variably possessing subdivided cervical scales [].

#### Postcrania

Thoroughly described and documented postcranial fossils are comparatively rare in the baenid fossil record [6]. In fact, Joyce & Lyson attribute all postcranial fossils that were originally ascribed to *B*. *arenosa*, *B*. *riparia*, and *B*. *sima* to the newly reinstated “*B*.” *affinis* [6]. Vertebral fossils are associated here with two new Uintan *B*. *arenosa* specimens: two cervical vertebrae of unknown position in UMNH VP 27535, and dorsal vertebrae (D8-10 and S1) in UMNH VP 27191 (Fig 3A and 3B). The smaller of the two cervical vertebra in *B*. *arenosa* specimen UMNH VP 27535 appears opisthocoelous (convex anteriorly, concave posteriorly) [Fig 3B]. This condition has been described in C8 of *C*. *undatum*, while the C2 and C3 of *C*. *undatum* are procoelous (concave anteriorly, convex posteriorly), and its C4 is amphicoelous (concave anteriorly and posteriorly) [8]. However, another possibility is that this surface in UMNH VP 27535 represents an intercentral body that has fused to the centrum. This condition is described in *Boremys pulchra* [57] and *Peckemys brinkman* [37], and likely represents a fused notochord [57].

Specimen UMNH VP 27535 also appears to have cervical ribs at multiple vertebral levels (Fig 3C and 3D). Reduced cervical ribs were present in the ancestral crown turtle lineage [58–60]. Within baenids, cervical ribs have been described in association with the atlas of *Boremys pulchra* [57], but are absent in *Arvinachelys* [36] and *Peckemys brinkman* [37]. The functional implications of these structures relate to the physical constraints they impose on neck retraction into the shell. Werneberg and colleagues [61] argue that cervical ribs limit the retraction mechanism by decreasing flexibility of the neck. This interpretation is consistent with previous studies suggesting that baenids may have had limited ability to retract their necks [2, 8, 62]. This restricted retraction was likely accompanied, however, by a muscular advantage. Cervical ribs serve as attachment sites for musculature of the neck [61], thus possibly providing *B*. *arenosa* a mechanical advantage in neck mobility. Thus, we infer that *B*. *arenosa*, despite having limited neck retraction capabilities, likely possessed powerful neck movement. The extensive bony roofing of the skull is consistent with the inability to pull the head within the shell (e.g., as in *Platysternon*).

### Phylogenetic and taxonomic implications of morphological variation

The morphological variation introduced into the hypodigms of *B*. *arenosa* and *C*. *undatum* by the new Uintan specimens suggested that a new analysis of baenid phylogenetic relationships was warranted. The resulting minimum length cladograms show weak support (52%) for a hypothesis of *B*. *arenosa* + *C*. *undatum* monophyly (Fig 17). However, a sister relationship of *C*. *undatum* + *Stygiochelys estesi* is supported in almost as many minimum length trees (48%). Thus, this relationship should not be considered full resolved, and as more specimens are recovered, the understanding can be refined. Uintan baenid monophyly has not been universally recovered in past studies of baenid phylogeny [6, 36]. However, it is consistent with many historical interpretations of relationships within the Baenidae [2, 37–38], and is also compatible with geotemporal distributions of the species. Interestingly, “*Baena*” *hayi*, *“Baena” affinis*, and “*Baena*” *escavada* all fell outside the clade of (*Stygiochelys* + (*B*. *arenosa* + *C*. *undatum*)). This finding provides further support for the paraphyletic status of the genus “*Baena*” and suggest that further taxonomic revision may be required. However, it should also be noted that we did not force a sister taxon relationship between any “*Baena*” species, and their apparently distant positions on the minimum trees may result from a comparative lack of data in some species (S1 File).

Recently, Joyce & Lyson [6] argued that “*Baena*” *affinis* [22] should be reinstated as a valid taxon. However, it is unlikely that the Uintan *Baena* specimens described here belong to this restored taxon. The characters suggested to define “*B*.” *affinis* include the lack of a sigmoidal humeral-extragular sulcus and mesoplastra without midline contact (some specimens), neither of which characterizes the new Uintan *Baena* specimens. Both groups share only traits that are also found in other eubaenines, including multiple cervical scales, thick neurocranial bone, and vertebrals II-IV that are longer than wide. “*Baena*” *affinis* autapomorphies, such as contact between the pectoral and a marginal scale and the presence of three inframarginals, cannot be conclusively assessed in the Uintan specimens due to incompleteness in these regions. If the Uintan specimens described here belong to “*B*.” *affinis*, they would represent the youngest “*B*.” *affinis* fossils yet described, since this taxon is documented only from the Bridgerian NALMA [8–9, 22].

In our phylogenetic analysis, *Baena arenosa* was found to be polymorphic for six characters, which was higher than any other taxon in the analysis. As additional specimens are discovered, it may become prudent to reassess whether multiple species exist within the *B*. *arenosa* hypodigm. However, there are currently few synapomorphies separating Uintan *B*. *arenosa* from those of other NALMAS. Therefore, for now, we adopt a conservative approach and retain it as a single species.

### Ontogenetic variation

The presence of subadult specimens of both *Baena arenosa* and *Chisternon undatum* in the sample allow an evaluation of the ontogenetic changes that occur during baenid development, as well as permit a more detailed exploration of the individual shell elements, which often become entirely fused in adults. In all subadult baenids described here, a midsagittal ridge courses antero-posteriorly along the dorsal surface of the neural row (Figs 10, 11 and 15). This mid-dorsal ridge is negligible or absent in all adult specimens. In the smaller subadults (UMNH VP 27540, UMNH VP 27544), the ridge is distinct and fans out into a triple ridge of three parallel elevations (Figs 11A, 11E and 15A). In the largest subadult specimen (UMNH VP 27537), a mid-dorsal ridge is present, but less pronounced (Fig 12A). This pattern suggests that during ontogenesis, the mid-dorsal ridge became increasingly diminished until it ceased to exist in adults.

This may be a simple developmental process; however, it is also possible that this phenomenon is the result of behavior. There is evidence that the mid-dorsal keel of modern bog turtles (genus *Glyptemys*) generally becomes worn down and obliterated in older adults as the result of years of digging and burrowing in boggy soils [63–64]. While *B*. *arenosa* and *C*. *undatum* are generally recovered from fluvial and lacustrine-dominated deposits and typically thought to have been predominantly aquatic, they have also been reconstructed as preferring riparian habitats (*B*. *arenosa*) and paludal environments (*C*. *undatum*) [26]. While their limb morphology shows no specialization for such digging, the water these baenids inhabited was turbid, and it is conceivable that their shells became smoothed by abrasion from suspended particles or pushing through abrasive vegetation. The interpretation that incremental reduction of the mid-dorsal ridge may result from abrasion is further supported by the observation that, in general, the carapace of Uintan subadult baenids tends to be more crenulated and textured, whereas those of adults tend to be smooth and lacking distinct ridges.

The patent sutures in the relatively complete plastral and midline carapace sections of *B*. *arenosa* subadult specimens UMNH VP 27540, UCMP 179495, UCMP 179520, and UMNH VP 27537 and *C*. *undatum* UMNH VP 27544 permit the comparison of individual shell elements (Figs 7, 11, 12 and 16). Most conspicuously, the shape and configurations of the unfused mesoplastra differ substantially between the two species. In the *C*. *undatum* subadult UMNH VP 27544, the mesoplastra are triangular, and narrow dramatically at the midline, resulting in a minimal midline articulation between the two sides (Fig 16C and 16D). The result is a chi (χ) configuration at the midline, and is the origin of the genus name [56]. The mesoplastra of *B*. *arenosa*, on the other hand, are much broader at the midline, resulting in a more extensive midline contact between the two bones (Figs 10C, 10D, 11A and 11B).

The shapes of the individual neural bones are also identifiable in the unfused immature specimens. While there are no distinct morphological differences in these bony elements between taxa, their configuration may provide insight into the ontogeny of dorsal carapace fusion. The anterior border of neurals II-V expands into two parasagittal anterior projections that create a wedge to accommodate the posterior projection of the preceding neural (Figs 7, 10A, 10B, 11A, 10B, 16A and 16B). The medial borders of the costals are hooked posteriorly, creating a tight articulation among contiguous costals and their adjacent costals. Thus, even in the subadults, there is an intimate connection among all the bony elements of the dorsal carapace. This intimate articulation may represent an early ontogenetic stage in the process of carapace fusion.

### Predation

Evidence of predation events and pressures shed insight onto the ecology of Uintan baenids. All of the relatively complete subadult baenid shells described here exhibit lesions, many of which appear characteristically like the classic puncture pits associated with carnivore bite marks [65]. Over a dozen deep puncture pits were observed on the internal surfaces of the carapace and plastron of *B*. *arenosa* subadult UMNH VP 27537, many of which pierced entirely through the bone (Fig 12). These lesions are large (4–5 mm in diameter) and rounded, with sloping margins of displaced compact and cancellous bone. Additionally, the shell of subadult UMNH VP 27540 is scattered with a number of small, round pits (Fig 11). These lesions are shallower than in UMNH VP 27537, and do not extend into the cancellous, diploë-like bone deep to the cortical layer. However, the sides are inclined, and the edges irregular, suggesting shallow compression punctures. The subadult *C*. *undatum* specimen UMNH VP 27544 displays two superficial compression punctures, one each on the external surfaces of the carapace and plastron (Fig 16). The margins of these pits are sharp and un-remodeled. These patterns of pathology are consistent with carnivore bite marks, as described in Hutchison and Frye [65], and almost certainly resulted from predation events. These pits appear to have occurred perimortem with little evidence of remodeling, suggesting that predation was likely the cause of death in these specimens.

Three adult baenid specimens also show signs of pitting, although these lesions are generally shallower and less numerous than in the subadult examples. *B*. *arenosa* adult UMNH VP 27546 displays a single large, round pit, approximately 7.5 mm in diameter (Fig 10D). The compact bone is displaced ventrally into the subadjacent cancellous layer. Some irregular bone, suggestive of osteological remodeling, extends into the void. The ventral plastron of *B*. *arenosa* specimen UMNH VP 27191 is scattered with several deep, round pits that perforate the bone (Fig 5C and 5D). Some of these lesions possess constrained margins with limited evidence of compression, and are unlikely to represent bite marks. However, a few of these pits show the characteristic signatures of compression puncture. The largest is oval-shaped and 6 x 8.5 mm in size. This lesion extends deeply into the cancellous bone, with displaced cortical walls that show evidence of compression. The adult *B*. *arenosa* UMNH VP 27539 displays a single shallow, irregularly bordered pit on the dorsal side of the carapace that does not extend through its extremely thick compact bone (Fig 6D). In all of these adult specimens, the punctures are sparse and do not perforate entirely through the shell, suggesting that they were unlikely the immediate cause of death in these specimens. Although such bite marks may not be directly fatal, the resulting open marrow spaces within the membranous bones of the shell can lead to destructive infections or septicemia, which could ultimately result in death [65].

Predation pressures appear to have been extensive for immature Uintan baenids, and a common cause of death at this developmental stage. For large-bodied adult baenids, however, predation was apparently less frequent. In addition to the generally decreased predation pressures that are typically afforded by a large body size, adult baenids also tend to have extremely thick shell bones due to their sutural fusion [6], which likely resulting in greater protection against predation. Moreover, the strong vertical buttresses would tend to pierce the palate of predators that crush the shell whole, such as crocodilians. In the Uintan NALMA, the primary predators of large aquatic turtles were likely crocodyliformes, (e.g., *Brachyuranochampsa*, *Borealosuchus*, *Crocodylus*, and *Allognathosuchus*). Smaller turtles, including subadult Baenids, were also likely preyed upon by mammalian predators, including carnivorans (e.g., *Miacis* and *Miocyon*), and possibly creodonts (e.g., *Limnocyon* and *Oxyaenodon*), and mesonychians like *Harpagolestes* [65]. We have observed similar bite mark evidence on other large aquatic Uintan turtles, such as *Echmatemys* (Smith, unpub. data).

### Geotemporal distribution

The ratio in our sample of 22 *B*. *arenosa* to 5 *C*. *undatum* specimens is almost identical to that described previously. Hutchison [26] noted that *B*. *arenosa* tends to be 2–3 times as abundant in assemblages as *C*. *undatum*. Baenids are more abundant in Ui2 strata (20/27 specimens = 74%) compared to Ui3 strata (7/27 specimens = 26%) [Table 1]. Interestingly, while previous studies have described these coeval taxa as occupying slightly different geographic distributions and habitat preferences, the present study found widespread overlap (Table 1). At two of the three Uintan localities from which new *C*. *undatum* specimens were recovered, we also found *Baena* specimens. However, we did observe a difference in the stratigraphic ranges from which the two species were recovered. *Baena arenosa* was discovered from low in the section (25 m) through the top of the section (366 m), thus ranging from Ui2-Ui3 [13, 35]. *C*. *undatum* was recovered only from localities low in the section, within Ui2 strata (25–87 m). However, the distribution of the latter could be an artifact of the relative rarity of this taxon and smaller sample size of *C*. *undatum* specimens recovered.

Perhaps unsurprisingly, many baenids derive from the Ui2 locality WU-22, aptly nicknamed “Terrapin Station”, and thereabouts [13]. In all localities from which we discovered *B*. *arenosa* and *C*. *undatum* specimens, we also found large quantities of the abundant geoemydid, *Echmatemys*. Uintan baenids are also often found with trionychids, which are a deep channel, highly aquatic family, often found in fluvial and even near-shore brackish environments. In fact, at one locality lower in the section, WU-8 (57-60m), trionychids outnumber baenids. At two localities (WU-34 and WU-223), baenids were also found in association with concentrations of the carettochelyid *Anosteira* (*Pseudanosteira*) *pulchra*. This pattern of co-occurrence with highly aquatic and semi-aquatic turtles reinforces the primarily aquatic habits of *B*. *arenosa* and *C*. *undatum*. Future studies extending broadly across turtle taxa can elucidate the nature of habitat and resource sharing among sympatric Uintan turtle communities. While collection bias can certainly not be excluded as affecting the higher proportion of baenids recovered from Ui2 strata, it is of note that many other turtle and mammal taxa have been recovered from Ui3 localities [15, 35, 66], but there is a notable paucity of baenid fossils. *Echmatemys*, *Anosteira*, and trionychids have all been recovered in high concentrations in Ui3, while baenid specimens dwindle in comparison.

One *B*. *arenosa* specimen, UMNH VP 27546, was recovered from the Uinta Formation/Duchesne River Formation contact (WU-123). This is the latest documented specimen of this species. Interestingly, the carapacial and plastral bones in this specimen are substantially thicker than in any of the other Uintan baenid specimens (Fig 10). Only through collecting above the Uinta Formation/Duchesne River Formation contact and exploring upsection into the Duchesne River Formation, will we be able to determine if increased shell thickness is a trait of late Uintan and (if found) early Duchesnean specimens of *Baena*.

The end of the Uintan NALMA was likely affected by the middle Eocene climatic optimum (MECO) hypothermal event, where a rather long period (500–750 ka) of increased warming has been detected in the marine record and on the North American continent in the Sage Creek Basin of Montana [16]. However, in the stratigraphic interval where the specimens reported here were recovered, preliminary oxygen isotope data indicate that precipitation was also increasing approaching the MECO hypothermal (Higgins, pers. comm). Habitat reconstructions based on faunal analyses have suggested that during the Uintan NALMA, there was a habitat shift away from the dense tropical forest typical of the earlier Eocene intervals to more open habitats, with trees likely clustering around fluvial systems [15]. Although global climates were cooling during the middle Eocene, it appears that local and regional climates were not radically effected [11, 14, 67–69].

By Ui2 time (~43Ma), within the study area in the eastern Uinta Basin, large river channels and deltaic sands prograding into the westward regressing Lake Uinta comprised the sediments where these turtles were recovered [18, 70–71]. The overlying Duchesne River Formation is typified by coarser, conglomeritic channel sandstones, indicative of a higher energy fluvial environment, and turtles are rarely recovered [72–73]. As the larger bodies of water in the Uinta Basin diminished in size and more fluvial environments (including rapid moving rivers) dominated the landscape, these large-bodied aquatic turtles may have struggled to adapt, and thus responded by decreasing body size. Other Uinta Basin turtle taxa, including Trionychia and Geoemydidae, show a similar trend towards decreasing body size in Ui3 [66, 74]. At this time, the large bodied aquatic baenids, the final surviving species of the abundant and speciose baenid radiation, have their final documented appearance in the fossil record.

## Supporting information

S1 File Nexus input file used for phylogenetic analyses. (TNT)

S1 Table Dimensions of individual cranial elements and features in *Baena* specimen UMNH VP 27535. (DOCX)

S2 Table Table indicating character states for *Baena arenosa* and *Chisternon undatum* included in the phylogenetic analysis. Character matrix was taken from Lyson et al., (2016), and codification changes for *B*. *arenosa* and *C*. *undatum* based on the new Uintan baenid specimens are highlighted in bold and indicated with an asterisk.
(DOCX)

## References

[1] Cope ED. Contributions to the history of the Vertebrata of the Lower Eocene of Wyoming and New Mexico, made during 1881. Proc Amer Phil Soc. 1882;20: 139–197.

[2] Gaffney ES. The systematics of the North America family Baenidae (Reptilia, Cryptodira). Bull Amer Mus Nat Hist. 1972a;147: 243–319.

[3] Hutchison JH. Turtle, crocodilian, and champosaur diversity changes in the Cenozoic of the northcentral region of western United States. Palaeogeogr Palaeocl. 1982;37: 147–167.

[4] Brinkman DB. Anatomy and systematics of *Plesiobaena antiqua* (Testudines: Baenidae) from the mid-Campanian Judith River Group of Alberta, Canada. J Vert Paleontol. 2003;23: 146–155.

[5] Lyson TL, Joyce WG. A new Baenid turtle from the Upper Cretaceous (Maastrichian) Hell Creek Formation of North Dakota and a preliminary taxonomic review of Cretaceous Baenidae. J Vert Paleontol. 2010;30: 394–402.

[6] Joyce WG, Lyson TL. A review of the fossil record of turtles in the clade Baenidae. Bull Peabody Mus Nat Hist. 2015;56(2): 147–183.

[7] Russell DA. Reptilian diversity and the Cretaceous-Tertiary transition in North America. Geol Assoc Canada 1975;13: 119–136.

[8] Hay OP. The fossil turtles of North America. Carnegie Institute Publ., 1908 no. 75, 1–568.

[9] Gilmore CW. The fossil turtles of the Uinta Formation. Mem Carnegie Mus. 1916;7: 101–161.

[10] Hutchison JH. Western North American reptile and amphibian record across the Eocene/Oligocene boundary and its climatic implications In: Prothero DR, Berggren WA, editors. Eocene-Oligocene climatic and biotic evolution. Princeton: Princeton University Press; 1992 pp 451–463.

[11] Prothero DR. Magnetic stratigraphy and biostratigraphy of the middle Eocene Uinta Formation, Uinta Basin, Utah In: Prothero DR, Emry RJ, editors. The terrestrial Eocene-Oligocene transition in North America. Cambridge: Cambridge University Press; 1996 pp. 75–119.

[12] Bohaty SM, Zachos JC. Significant Southern Ocean warming event in the late middle Eocene. Geology 2003;31(11): 1017–1020.

[13] Townsend KE, Friscia AR, Rasmussen DT. Stratigraphic distribution of upper Middle Eocene vertebrate localities in the eastern Uinta Basin, Utah, with comment on Uintan biostratigraphy. Mount Geol. 2006;43: 115–134.

[14] Zachos JC, Dickens GR, Zeebe RE. An early Cenozoic perspective on greenhouse warming and carbon-cycle dynamics. Nature 2008;451(7176): 279–283. doi: 10.1038/nature06588 18202643 10.1038/nature06588

[15] Townsend KE, Rasmussen DT, Murphey PC, Evanoff E. Middle Eocene habitat shifts in the North American western interior: A case study. Palaeogeogr Palaeocl 2010;297: 144–158.

[16] Methner K, Mulch A, fiebig J, Wacker U, Gerdes A, Graham SJ, et al Rapid Middle Eocene temperature change in western North America. Earth Planet Sci Lett. 2016;450: 132–139.

[17] Ryder RT, Fouch TD, and Elison JH. Early Tertiary sedimentation in the western Uinta Basin, Utah. Geol Soc Amer Bull 1976;87: 496–512.

[18] Davis SJ, Wiegand BA, Carroll AR, Chamberlain CP. The effect of drainage reorganization on paleoaltimetry studies: An example from the Paleogene Larmide foreland. Earth Planet Sci Lett. 2008;275: 258–268.

[19] Chamberlain CP, Mix HT, Mulch A, Hren MT, Kent-Corson ML, Davis SJ, et al The Cenozoic climatic and topographic evolution of the North American Cordillera. Am J Sci. 2012;312: 213–262.

[20] Leidy J. Description of *Emys jeanesi* n. sp., *Emys haydeni* n. sp., *Baena arenosa* n. g. n. sp., and *Saniwa ensidens* n. g. n. sp. Proc Acad Nat Sci Phila. 1870:123–124.

[21] Leidy J. On a new genus of extinct turtles. Proc Acad Nat Sci Phila. 1872:162.

[22] Leidy J. Remarks on fossil vertebrates from Wyoming. Proc Acad Nat Sci Phila. 1871:228–229.

[23] Auffenberg WA. Fossil turtles of the genus *Terrapene* in Florida. Florida St Mus Bull 1958;3: 53–92.

[24] Milstead WW. Studies on the evolution of the box turtles (genus *Terrapene*). Florida St Mus Bull. 1969;14: 1–113.

[25] Milstead WW, Tinkle DW. *Terrapene* of western Mexico with comments on the species groups in the genus. Copeia 1967:180–187.

[26] Hutchison JH. Determinate growth in the Baenidae (Testudines): taxonomic, ecologic, and stratigraphic significance. J Vert Paleontol 1987;3: 148–151.

[27] Hillson S, Fitzgerald C, Flinn H. Alternative dental measurements: proposals and relationships with other measurements. Am J Phys Anthropol. 2005;126: 413–426. doi: 10.1002/ajpa.10430 15386233 10.1002/ajpa.10430

[28] Wood HE, Chaney RW, Clark J, Colbert EH, Jepsen GL, Reeside JB, et al Nomenclature and correlation of the North American continental Tertiary. GSA Bull. 1941;52: 1–48.

[29] Rasmussen DT, Townsend KE. New small-bodied mammals from the Uinta Formation, Uinta Basin, Utah, contrast with the coeval small mammals of California. J Vert Paleontol 1995;15(3S): 49.

[30] Rasmussen DT. A new Middle Eocene omomyine primate from the Uinta Basin, Utah. J Hum Evol. 1996;31: 75–87.

[31] Rasmussen DT, Conroy GC, Friscia AR, Townsend KE, Kinkle MD. Mammals of the middle Eocene Uinta Formation In: Gillette DD, editor. Vertebrate paleontology in Utah. Salt Lake City: Utah Geological Survey; 1999 pp. 402–420.

[32] Townsend KE. 2004. Stratigraphy, paleoecology, and habitat change in the Middle Eocene of North America. Ph.D. dissertation, Washington University.

[33] Osborn HF. Fossil mammals of the Uinta Basin. Expedition of 1894. Bull Amer Mus Nat Hist 1895;7: 71–105.

[34] Osborn HF. The Titanotheres of ancient Wyoming, Dakota, and Nebraska. USGS Monograph 1929;1: 1–701.

[35] Gunnell GF, Murphey PC, Stucky RK, Townsend KE, Robinson P, Zonneveld J-P, et al Biostratigraphy and biochronology of the latest Wasatchian, Bridgerian, and Uintan North American Land Mammal “Ages”. Mus North Ariz Bull. 2009;65: 279–330.

[36] Lyson TL, Joyce WG, Lucas SG, Sullivan RM. A new baenid turtle from the early Paleocene (Torrejonian) of New Mexico and a species-level phylogenetic analysis of Baenidae. J Paleontol. 2016;90: 305–316. doi: 10.1017/jpa.2016.47

[37] Lyson TL, Joyce WG. A revision of *Plesiobaena* (Testudines: Baenidae) and an assessment of Baenid ecology across the K/T Boundary. J Paleontol. 2009;83(6): 833–853.

[38] Lyson TR, Joyce WG. A new species of *Palatobaena* (Testudines: Baenidae) and a maximum parsimony and Bayesian phylogenetic analysis of Baenidae. J Paleontol. 2009b;83: 457–470.

[39] Lyson TR, Joyce WG. Cranial anatomy and phylogenetic placement of the enigmatic turtle Compsemys victa Leidy, 1856. J Paleontol. 2011;85: 789–801.

[40] Larson DW, Longrich NR, Evans DC, Ryan MJ. A new species of *Neurankylus* from the Milk River Formation (Cretaceous: Santonian), Alberta, and a revision of *N*. *eximius* In: Brinkman DB, Holroyd PA, Gardner JD, editors. Morphology and evolution of turtles: Proceedings of the Gaffney Turtle Symposium in honor of Eugene S. Gaffney. New York: Springer; 2012 pp. 389–405.

[41] Goboloff PA, Catalano SA. TNT version 1.5, including a full implementation of phylogenetic morphometrics. Cladistics 2016;32: 221–238. 10.1111/cla.12160 34727670

[42] Batsch JGC. Versuch einer Anleitung, zur Kenntni und Geschichte der Thiere und Mineralien. Jena: Akademische Buchhandlung; 1788.

[43] Gaffney ES. A phylogeny and classification of the higher categories of turtles. Bull Amer Mus Nat Hist. 1975;155: 387–436.

[44] Williams EE. Variation and selection in the cervical central articulations of living turtles. Bull Amer Mus Nat Hist 1950;94: 511–561.

[45] Gaffney ES, Meylan PA. A phylogeny of turtles In: Benton MJ, editor. The phylogeny and classification of the Tetrapods, Volume 1. Amphibians, reptiles, birds. Systematics Association Special Volume No. 35A. Oxford: Clarendon Press; 1988 pp. 157–291.

[46] Gaffney ES. Comparative cranial morphology of recent and fossil turtles. Bull Amer Mus Nat Hist. 1979;164: 69–376.

[47] Gaffney ES. An illustrated glossary of turtle skull nomenclature. Amer Mus Novitates 1972b;2486: 1–33.

[48] Gaffney ES. Cranial morphology of the Baenid turtles. Amer Mus Novitates 1982a;2737: 1–22.

[49] Hay OP. On some fossil turtles belonging to the Marsh collection in the Yale University Museum. Am J Sci. 1904;18: 261–276.

[50] Schumaker G-H. Beiträge zur Kiefermuskulatur der Schildkröten. I. Mitteilung. Wiss. Zeitschr, Univ. Greifswald, Jahrgang 3, Math.-Naturwiss. 1954: 457–518.

[51] Schumaker G-H. Beiträge zur Kiefermuskulatur der Schildkröten. II. Mitteilung. Wiss. Zeitschr, Univ. Greifswald, Jahrgang 4, Math.-Naturwiss. 1955a: 501–518.

[52] Schumaker G-H. Beiträge zur Kiefermuskulatur der Schildkröten. III. Mitteilung. Wiss. Zeitschr, Univ. Greifswald, Jahrgang 4, Math.-Naturwiss. 1955b: 559–587.

[53] Schumaker G-H. Morphologische Studie zum Gleitmachanismus des M. adductor mandibularis externus bei Schildkröten. Ana Anz. 1956;103: 1–12. 13314129

[54] Gaffney ES. The lower jaws of Baenid turtles. Amer Mus Novitates 1982b;2749: 1–10.

[55] Hutchison JH. Guide to the Uinta turtles. Unpub. Field guide. 1996.

[56] Leidy J. Contributions to the extinct vertebrate fauna of the Western Territories. Report of the U.S. Geological Survey of the Territories 1873;1: 14–358.

[57] Brinkman DB, Nicholls EL. Anatomy and relationships of the turtle *Boremys pulchra* (Testudines: Baenidae). J Vert Paleontol. 1991;11: 302–315.

[58] Joyce WG. Phylogenetic relationships of Mesozoic turtles. Bull Peabody Mus Nat Hist. 2007;48: 3–102.

[59] Anquetin J. Reassessment of the phylogenetic interrelationships of basal turtles (Testudinata). J Syst Palaeontol. 2012;10: 3–45.

[60] Sterli J, de la Fuente MS. New evidence from the Palaeocene of Patagonia (Argentina) on the evolution and palaeo-biogeography of Meiolaniformes (Testudinata, new taxon name). J Syst Palrontol 2013;11: 835–852.

[61] Werneburg I, Hinz JK, Joyce WG. Embryonic remnants of intercentra and cervical ribs in turtles. Biol Open 2013;2: 1103–1107. doi: 10.1242/bio.20135439 24244846 10.1242/bio.20135439 PMC3828756

[62] Werneburg I, Maier W, Gumpenberger M, Volpato V, Natchev N, Joyce WG. Modeling neck mobility in fossil turtles. J Exp Zool B- Mol Devel Evol. 2014;324: 230–243. 24497449 10.1002/jez.b.22557

[63] Bury BR. Review of the ecology and conservation of the bog turtle, *Clemmys muhlenbergii*. U.S. Department of Interior, U.S. Fish and Wildlife Service Special Scientific Report 1979;219: 1–9.

[64] Somers AB, Bridle KA, Herman DW, Nelson AB. The restoration and management of small wetlands of the mountains and piedmont in the Southeast: A manual emphasizing endangered and threatened species habitat with a focus on bog turtles. Greensboro: Natural Resources Conservation Service; 2000.

[65] Hutchison JH, Frye FL. Evidence of pathology in early Cenozoic turtles. PaleoBios 2001;21: 12–19.

[66] Adrian B, Hutchison JH, Townsend KE. New reports and distribution of rare turtles in the Uinta Formation, including specimens from Carettochelyidae, Planetochelyidae, Testudinidae, Geoemydidae, and Baenidae. J Vert Paleontol. 2016;36S: 86.

[67] Zachos J, Shackleton N, Revenaugh J, Palike H, Flower B. Climate response to orbital forcing across the Oligocene-Miocene boundary. Science 2001;292(5515): 274–278. doi: 10.1126/science.1058288 11303100 10.1126/science.1058288

[68] Barbieri R, Benjamini C, Monechi S, Reale V. Stratigraphy and benthic foraminiferal events across the Middle-Late Eocene transition in western Negev (Israel) In: Prothero DR, Nesbitt E, Ivany L, editors. From greenhouse to icehouse: The marine Eocene-Oligocene transition. New York: Columbia University Press; 2003 pp. 452–470.

[69] Hurley JV, Fluegeman RH. Late-middle Eocene glacio-eustasy: Stable isotopes and foraminifera from the Gulf Coast Plain In: Prothero DR, Nesbitt E, Ivany L, editors. From greenhouse to icehouse: The marine Eocene-Oligocene transition. New York: Columbia University Press; 2003 pp. 223–231.

[70] Bryant B, Naeser CW, Marfin RF, Mehnert HH. Upper Cretaceous and Paleogene sedimentary rocks and isotopic ages of Paleogene tuffs, Uinta Basin, Utah. USGS Bull. 1989;1787-J.

[71] Smith ME, Carroll AR, Singer BS. Synoptic reconstruction of a major ancient lake system: Eocene Green River Formation, western United States. Geo Soc Amer Bull. 2008;120: 54–84.

[72] Clark J. A new turtle from the Duchesne Oligocene of the Uinta Basin, northeastern Utah: Ann Carnegie Mus. 1932;21: 131–133.

[73] Andersen DW and Picard MD. Stratigraphy of the Duchesne River Formation (Eocene-Oligocene?), northern Uinta Basin, northeastern Utah. Utah Geol Min Soc Bull. 1972;97: 1–23.

[74] Jager DM, Smith HF, Hutchison JH, Jorge K, Adrian B, Townsend KE. 3D epiplastral, geographic, and body size variation in *Echmatemys*, a geoemydid turtle form the Uinta Formation, Uinta Basin, Utah, USA. J Vert Paleontol. 2016;36S: 160.

