# Supplementary material for: Morphological variation, phylogenetic relationships, and geographic distribution of the Baenidae (Testudines), based on new specimens from the Uinta Formation (Uinta Basin), Utah (USA)
Source: PLoS One. 2017 Jul 7;12(7):e0180574. doi: 10.1371/journal.pone.0180574 (PMC5501565; doi:10.1371/journal.pone.0180574)
Supplement: S1 Table — (DOCX) [file pone.0180574.s002.docx]

**Suppl. Table 1.** Dimensions of individual cranial elements and features in *Baena* specimen UMNH VP 27535

Basioccipital: 10.6 mm wide x 7.4 mm long (including condylus occipitalis)

Condylus occipitalis: 7.7 mm wide x 5.2 mm long

Basisphenoid: 13.1 mm wide (at widest point) x 10.7 mm long

Frontal (L): 12.3 mm long

**Foramina:**

Foramen magnum (est.): 7.4 mm wide

Foramen nervi hypoglossi: Internal= 1.2 mm (caudal foramen) and 0.6 mm (ventral foramen)

External= 0.9 mm (caudal foramen) and 0.8 mm (ventral foramen)

Canalis hypoglossi: 0.7 mm diameter, 4.3 mm long

Foramina posterius canalis carotici: 1.3 mm diameter

Foramina anterius canalis carotici interni: 0.8 mm diameter

Foramina jugulare posterius: 3.3 mm wide x 2.3 mm high

Foramen nervi abducentis: 0.5 mm diameter

Foramen supraorbitale: 0.6 mm diameter

Foramen alveolare superius: 1.1 mm diameter

Incisura columellae auris: 3.6 mm minimum (broken)

Foramen chorda tympani inferius: L- 0.8 mm, R- 0.9 mm

Foramen stapedio-temporale (broken): est. 1.3 mm

Foramen intermandibularis caudalis (prearticular): 1.3 mm high, open posteriorly

**Tubercles and Bony Features:**

Basis tuberculi basalis: 0.6 mm

Sella turcica: 4.5 mm wide and 2 mm deep

Fossae on ventral side of nasal aperture: 4.8 mm wide x 5.8 mm long

Sulcus olfactorius: 10.7 mm wide x 7.9 mm long x 4.1 mm deep

Cavum cranii: 9.0 mm wide at its cranial-most margin, widens posteriorly beyond the extent of the fragmentation. Thus, maximum width and length are not measurable.

Alveolar process (maxilla): 4.8 mm from base of processus palatinis to margin of triturating surface

Processus articularis (quadrate): L- 3.8 mm long, 10.3 mm wide. R- 3.2 mm long, 10.3 mm wide

Articular surface/fossa on processus articularis (quadrate): Left- 9.3 mm wide (ML), 5.7 mm long (AP), 1.4 mm deep. Right- 8.3 mm wide, 5.2 mm long, 1.5 mm deep

Canalis cavernosus: ~1.4 mm diameter

Rostrum basisphenoidale: 5.4 mm long

Canali nervi vidiani: 2 mm wide

Processus coronoideus (coronoid): 7.1 mm

Area articularis mandibularis: Articular: 6.4 mm wide, 7.9 mm long (tall).

Process for articulation of M. depressor mandibulae (articular): 2.3 mm long
