# Supplementary material for: Morphological variation, phylogenetic relationships, and geographic distribution of the Baenidae (Testudines), based on new specimens from the Uinta Formation (Uinta Basin), Utah (USA)
Source: PLoS One. 2017 Jul 7;12(7):e0180574. doi: 10.1371/journal.pone.0180574 (PMC5501565; doi:10.1371/journal.pone.0180574)
Supplement: S2 Table — Character matrix was taken from Lyson et al., (2016), and codification changes for B. arenosa and C. undatum based on the new Uintan baenid specimens are highlighted in bold and indicated with an asterisk. (DOCX) [file pone.0180574.s003.docx]

**Suppl Table 2.** Table indicating character states for *Baena arenosa* and *Chisternon undatum* included in the phylogenetic analysis. Character matrix was taken from Lyson et al., (2016), and codification changes for *B. arenosa* and *C. undatum* based on the new Uintan baenid specimens are highlighted in bold and indicated with an asterisk.

|  | 1 | 2 | 3 | 4 | 5 | 6 | 7 | 8 | 9 | 10 | 11 | 12 | 13 | 14 | 15 | 16 | 17 | 18 | 19 | 20 | 21 | 22 | 23 |
| --- | --- | --- | --- | --- | --- | --- | --- | --- | --- | --- | --- | --- | --- | --- | --- | --- | --- | --- | --- | --- | --- | --- | --- |
| New coding with Uintan baenids |  |  |  |  |  |  |  |  |  |  |  |  |  |  |  |  |  |  |  |  |  |  |  |
| *B. arenosa* | 1 | 0 | 1 | 0 | 0 | 1 | 1 | 0 | 0 | 1 | 1 | ? | ? | 1 | 1 | 0 | 1 | 0 | 0 | 0 | ? | ? | ? |
| *C. undatum* | 1 | 0 | 1 | 0 | 1 | **1*** | [0 1] | 0 | 0 | 1 | 1 | 2 | ? | 1 | 1 | 0 | 1 | 0 | 0 | 0 | 1 | 1 | 1 |
| Original coding (Joyce et al. 2016) |  |  |  |  |  |  |  |  |  |  |  |  |  |  |  |  |  |  |  |  |  |  |  |
| *B. arenosa* | 1 | 0 | 1 | 0 | 0 | 1 | 1 | 0 | 0 | 1 | 1 | ? | ? | 1 | 1 | 0 | 1 | 0 | 0 | 0 | ? | ? | ? |
| *C. undatum* | 1 | 0 | 1 | 0 | 1 | 2 | [0 1] | 0 | 0 | 1 | 1 | 2 | ? | 1 | 1 | 0 | 1 | 0 | 0 | 0 | 1 | 1 | 1 |

|  | 24 | 25 | 26 | 27 | 28 | 29 | 30 | 31 | 32 | 33 | 34 | 35 | 36 | 37 | 38 | 39 | 40 | 41 | 42 | 43 | 44 | 45 | 46 |
| --- | --- | --- | --- | --- | --- | --- | --- | --- | --- | --- | --- | --- | --- | --- | --- | --- | --- | --- | --- | --- | --- | --- | --- |
| New coding with Uintan baenids |  |  |  |  |  |  |  |  |  |  |  |  |  |  |  |  |  |  |  |  |  |  |  |
| *B. arenosa* | ? | 0 | 0 | **1*** | 1 | **1*** | ? | ? | 0 | ? | 1 | ? | 1 | ? | 0 | 1 | 0 | 0 | 1 | 0 | 1 | 0 | **0*** |
| *C. undatum* | 0 | 0 | 0 | 1 | 1 | 0 | 1 | ? | 1 | 1 | 1 | 0 | 0 | 2 | 0 | 1 | **[0 1]*** | 0 | 0 | 0 | 1 | 0 | 1 |
| Original coding (Joyce et al. 2016) |  |  |  |  |  |  |  |  |  |  |  |  |  |  |  |  |  |  |  |  |  |  |  |
| *B. arenosa* | ? | 0 | 0 | ? | 1 | ? | ? | ? | 0 | ? | 1 | ? | 1 | ? | 0 | 1 | 0 | 0 | 1 | 0 | 1 | 0 | ? |
| *C. undatum* | 0 | 0 | 0 | 1 | 1 | 0 | 1 | ? | 1 | 1 | 1 | 0 | 0 | 2 | 0 | 1 | 1 | 0 | 0 | 0 | 1 | 0 | 1 |

|  | 47 | 48 | 49 | 50 | 51 | 52 | 53 | 54 | 55 | 56 | 57 | 58 | 59 | 60 | 61 | 62 | 63 | 64 | 65 | 66 | 67 | 68 | 69 |
| --- | --- | --- | --- | --- | --- | --- | --- | --- | --- | --- | --- | --- | --- | --- | --- | --- | --- | --- | --- | --- | --- | --- | --- |
| New coding with Uintan baenids |  |  |  |  |  |  |  |  |  |  |  |  |  |  |  |  |  |  |  |  |  |  |  |
| *B. arenosa* | ? | **0*** | 1 | - | 0 | 0 | 0 | 0 | **0*** | 2 | 1 | 0 | 1 | 0 | 0 | 0 | 1 | 1 | **[0 1]*** | 1 | 1 | **1*** | ? |
| *C. undatum* | 1 | **0*** | 1 | - | 1 | 1 | ? | 0 | 1 | 2 | **1*** | 0 | 1 | 0 | 0 | 1 | 1 | **[0 1]*** | 1 | 1 | 1 | 1 | ? |
| Original coding (Joyce et al. 2016) |  |  |  |  |  |  |  |  |  |  |  |  |  |  |  |  |  |  |  |  |  |  |  |
| *B. arenosa* | ? | ? | 1 | - | 0 | 0 | 0 | 0 | ? | 2 | 1 | 0 | 1 | 0 | 0 | 0 | 1 | 1 | 1 | 1 | 1 | ? | ? |
| *C. undatum* | 1 | ? | 1 | - | 1 | 1 | ? | 0 | 1 | 2 | ? | 0 | 1 | 0 | 0 | 1 | 1 | 0 | 1 | 1 | 1 | 1 | ? |
